# A Phylogenetic Study of SPBP and RAI1: Evolutionary Conservation of Chromatin Binding Modules 

**DOI:** 10.1371/journal.pone.0078907

**Published:** 2013-10-18

**Authors:** Sagar Darvekar, Cecilie Rekdal, Terje Johansen, Eva Sjøttem

**Affiliations:** Molecular Cancer Research Group, Department of Medical Biology, University of Tromsø, Tromsø, Norway; Universität Stuttgart, Germany

## Abstract

Our genome is assembled into and array of highly dynamic nucleosome structures allowing spatial and temporal access to DNA. The nucleosomes are subject to a wide array of post-translational modifications, altering the DNA-histone interaction and serving as docking sites for proteins exhibiting effector or “reader” modules. The nuclear proteins SPBP and RAI1 are composed of several putative “reader” modules which may have ability to recognise a set of histone modification marks. Here we have performed a phylogenetic study of their putative reader modules, the C-terminal ePHD/ADD like domain, a novel nucleosome binding region and an AT-hook motif. Interactions studies *in vitro* and in yeast cells suggested that despite the extraordinary long loop region in their ePHD/ADD-like chromatin binding domains, the C-terminal region of both proteins seem to adopt a cross-braced topology of zinc finger interactions similar to other structurally determined ePHD/ADD structures. Both their ePHD/ADD-like domain and their novel nucleosome binding domain are highly conserved in vertebrate evolution, and construction of a phylogenetic tree displayed two well supported clusters representing SPBP and RAI1, respectively. Their genome and domain organisation suggest that SPBP and RAI1 have occurred from a gene duplication event. The phylogenetic tree suggests that this duplication has happened early in vertebrate evolution, since only one gene was identified in insects and lancelet. Finally, experimental data confirm that the conserved novel nucleosome binding region of RAI1 has the ability to bind the nucleosome core and histones. However, an adjacent conserved AT-hook motif as identified in SPBP is not present in RAI1, and deletion of the novel nucleosome binding region of RAI1 did not significantly affect its nuclear localisation.

## Introduction

Packaging genetic information into nucleosome structures represents barrier to any cellular process that needs to access DNA, including replication, repair, recombination, transcription, gene silencing and RNA maturation. There exist two main regulatory mechanisms, chromatin modifying activity and chromatin remodeling activity, which increase accessibility of genome to DNA binding proteins. Chromatin modifying activities lead post-translational modifications (PTMs) on histone tails and histone core, whereas chromatin remodeling activities modify non-covalent interactions between DNA and histones (reviewed in 1). The PTMs of histones and other chromatin proteins, form dynamic platforms that are able to assemble the machineries involved in DNA metabolism and to recruit chromatin modifying activities. Important players are chromatin binding proteins containing so-called “reader” modules recognizing specific PTMs on the histones. The “reader” proteins are often inherent in large multi-protein complexes classified as transcriptional co-activators [2-4]. Co-activator complexes generally exhibit several “reader” and effector modules, linking in a combinatorial way particular PTM marks to defined downstream events. A number of histone “reader” modules with distinct structural folds have been identified and characterized [reviewed in 5], such as bromo, chromo, Tudor, PWWP, MBT, plant Agenet and plant homeodomain (PHD) fingers. Recently several structural studies highlighting the combinatorial read out of multiple histone marks by single or tandemly arranged reader modules are reported [6-17]. 

**Figure 1:**
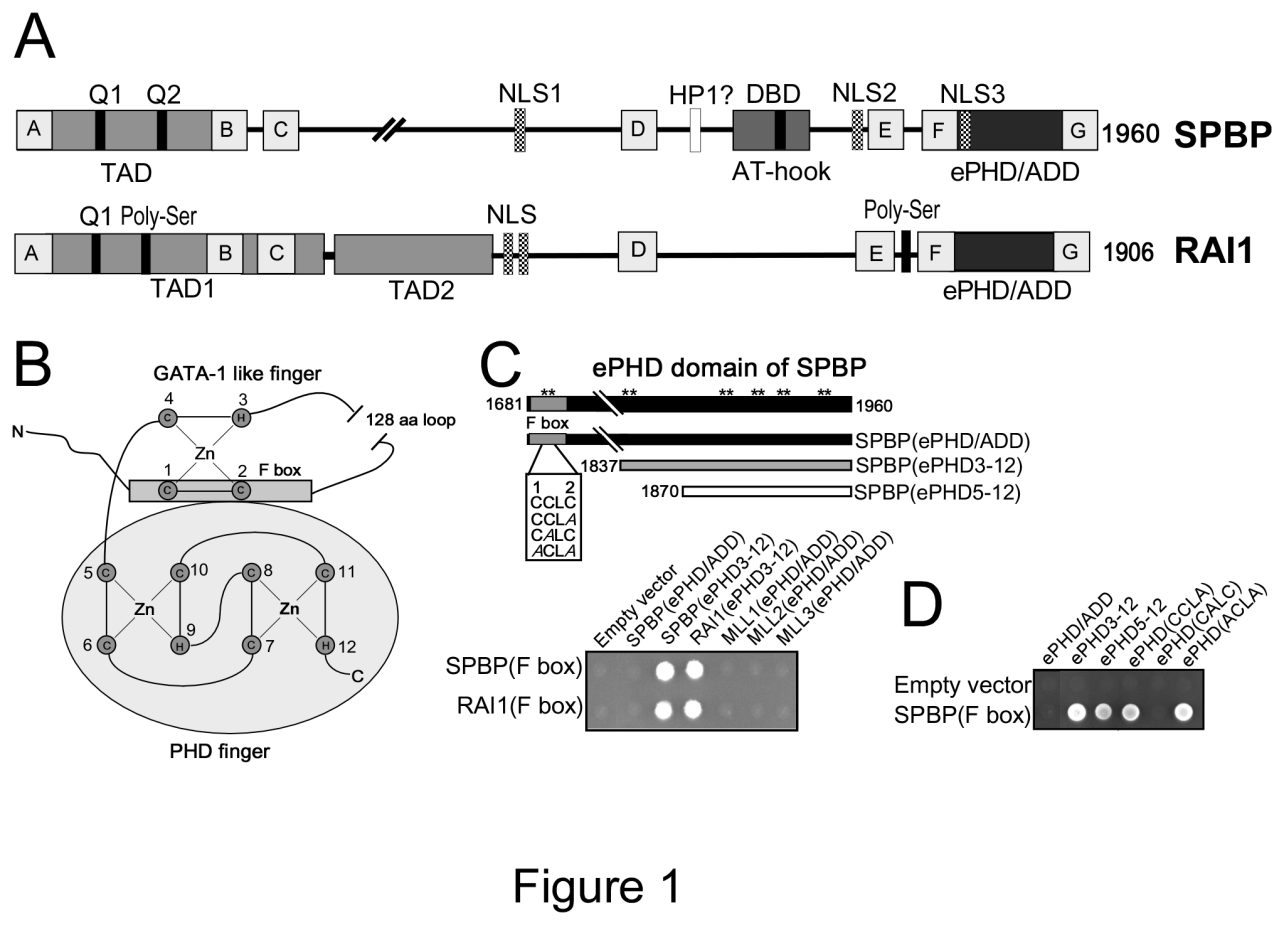
The F-boxes of SPBP and RAI1 interact with their atypical PHD fingers. (**A**) Schematic illustration of domain structures of human SPBP (1960aa) and RAI1 (1906aa). TAD: trans-activation domain, NLS: nuclear localization signals, DBD: DNA-binding domain, ePHD/ADD: extended plant homeodomain, Q1/Q2: glutamine-rich stretches, grey boxes A to G: represents evolutionary conserved regions with strong homology between SPBP and RAI1. (**B**) A cartoon depicting a model of the ePHD domain of SPBP. In this model the F box interacts with the PHD domain of SPBP to form an ePHD/ADD domain containing two interacting zinc-finger modules. The interleaved zinc-ligation topology of the PHD domain is shown. Abbreviations: Zn, zinc; C, cysteine; H, histidine; N, N-terminal end; C, C-terminal end. (**C**) A schematic presentation of the ePHD/ADD domain of SPBP is shown. Asterisks mark Cys and His residues that may serve as zinc ligands. The F box is indicated in dark grey. The N- and C-terminal amino acid positions demarcating the SPBP ePHD/ADD domain are shown. Truncated versions of the ePHD/ADD domains of SPBP and RAI1, containing zinc-ligands 3-12 interact with their own and the others F box, as revealed by two-hybrid analysis. The F boxes of both proteins were fused to GAL4-AD and tested towards GAL4-DBD constructs of SPBP(ePHD), SPBP(ePHD3-12), RAI1(ePHD3-12), MLL1(ePHD), MLL2(ePHD) and MLL3(ePHD). Interactions are indicated by growth on QDO medium. Yeast cell suspensions were spotted onto plates and allowed to grow at 30°C for 3 days. (**D**) Deletion- and point-mutational analysis of the ePHD/ADD domain of SPBP using the two-hybrid interaction system. SPBP (ePHD/ADD) (black), SPBP (ePHD3-12) (grey) and the PHD finger, named SPBP (ePHD5-12) (white) were fused to GAL4-DBD and tested for interaction with the F box of SPBP fused to GAL4-AD. In addition, three constructs obtained by site-directed mutagenesis, generating mutations in the putative zinc-ligands 1 and 2 in the F box of SPBP(ePHD/ADD) were fused to GAL4-DBD, and tested for interaction to the SPBP F box fused to GAL4-AD. Cysteine(s) are mutated to alanine(s) as indicated in (C).

 Stromelysin-1 PDGF (platelet-derived growth factor)-responsive element binding protein (SPBP; also named as TCF20) and Retinoic Acid Induced protein (RAI1) are two nuclear chromatin binding multidomain proteins containing seven regions with strong sequence similarity, in addition to similar domain organization (Figure 1 A). Their genome structures suggest that they probably have evolved by gene duplication [18]. Both proteins are involved in modulation of transcriptional activity. However, in our studies SPBP seems to act as a transcriptional activator, while RAI1 acts more as a transcriptional repressor [19]. Notably, SPBP exhibit several regions with ability to bind DNA and/or nucleosomes: i) an ePHD/ADD domain (consisting of an atypical PHD domain and a putative GATA-1 like zinc-finger both with the potential to bind chromatin) ii) a novel nucleosome binding region [20] and iii) a DNA binding domain with an AT-hook motif [21] (Figure 1A). Additionally, the ePHD/ADD domain contributes in protein-protein interactions [19]. Also the ePHD/ADD domain of RAI1 has the ability to bind nucleosomes [20]. However, other nucleosome or DNA binding activities in RAI1 has so far not been characterized. Interestingly, the ePHD/ADD domain of SPBP and RAI1 contains a long loop region between zinc ligands 2 and 3. Such long loop region is not found in other proteins containing ePHD/ADD domains, makes it a hallmark of SPBP and RAI1. 

 SPBP is expressed in most tissues ([21] and www.proteinatlas.com), and was originally found to be involved in transcriptional activation of Matrix Metalloproteinase 3 promoter via a specific DNA sequence [22]. Later, SPBP is shown to act as a transcriptional coactivator enhancing the transcriptional activity of certain transcription factors and cofactors [21,23]. SPBP is also shown to repress the transcriptional activity of estrogen receptor α (ERα) [24]. Over-expression of SPBP inhibited the proliferation of an ERα-dependent breast cancer cell line [24]. RAI1 shows a more restricted expression pattern than SPBP, mainly detected in kidney, endocrine glands, liver and neuronal cells (www.proteinatlas.com). RAI1 is clinically associated with Smith-Magenis Syndrome [25], Potocki-Lupaski Syndrome [26] and schizophrenia [27]. RAI1 is also described to be related with non-syndromic autism [28] and spinocerebellar ataxia type 2 [29]. So far, no clinically related disease has been connected to mutations or deletions in SPBP.

In this study, we utilized *in vitro* and yeast two-hybrid interaction studies to investigate whether the putative cysteine-rich motif preceding the atypical PHD domain of SPBP has the ability to form a GATA-1 like structure. On the basis of the predicted structural organization of the SPBP and RAI1 ePHD/ADD domains, the conservation of SPBP and RAI1 like sequences were analyzed using phylogenetic constructions. Our findings indicate the existence of both genes in vertebrates, one gene in lancelet and insects, while no genes were identified in plants, fungi or lower eukaryotes. Furthermore, the novel nucleosome binding region with the adjacent AT-hook motif was highly conserved within the SPBP proteins, while the AT-hook motif was absent in RAI1. However, in *vitro* pull down assays suggested that the region of RAI1 similar to the novel nucleosome binding region of SPBP, had the ability to bind nucleosomes and histones. Hence, organization of two independent chromatin binding regions in the transcriptional coregulators SPBP and RAI1 seems to be conserved in evolution. 

## Materials and Methods

### Plasmid constructs

Plasmids used in this work are listed in Supporting information, Table S1, and specific PCR primers used for amplification of cDNAs are listed in Supporting Information, Table S2. All PCR reactions were performed with the *Pfu* polymerase according to the instructions from the manufacturer (Stratagene). Plasmid constructs were verified by DNA sequencing using the BigDye sequencing kit (Applied Biosystems). Most of the expression plasmids were generated using the Gateway Cloning System (Invitrogen). The cDNA constructs were either subcloned using restriction enzymes, or transferred via the BP reaction of attB-flanked PCR products, into Gateway entry/donor vectors. Expression clones were made as described in the Gateway cloning technology instruction manual (Invitrogen). Mutagenesis of plasmid DNA was performed using the QuikChange site-directed mutagenesis kit (Stratagene, La Jolla, CA). Oligonucleotides for PCR, DNA sequencing and mutagenesis reactions were obtained from Invitrogen, Sigma Aldrich and Eurogentec. All other constructs including full length SPBP and RAI1 constructs are described previously [19-21] 

### Yeast two-hybrid screening and interaction assays

The yeast two-hybrid screening and interaction assays were carried out as described previously [19,30].

### Antibodies

The following primary antibodies were used in this study: anti-histone H3 (ab1791, Abcam) (1:1000), anti-GST (glutathione S-transferase) (Santa Cruz Biotechnology) (1:1000) and secondary antibody HRP (horseradish peroxidase)-conjugated goat anti-rabbit IgG (1:1000). 

### Cell culture

HeLa cells were grown in Dulbecco’s Modified Eagle Medium (DMEM) supplemented with 10% FBS (fetal bovine serum), 100 units/ml penicillin and 100 µg/ml streptomycin.

### Bioinformatics

The Blastp program was used to search with the ePHD/ADD domain (aa1690-1939) of SPBP for similar regions in other species (http://www.uniprot.org/). The alignment of the deduced amino acid sequences were carried out using ClustalW multiple alignment software. The online server of PhyML 3.0 was used to study phylogenetic evolution of ePHD/ADD domain of SPBP (http://www.atgc-montpellier.fr/phyml/). The phylogenetic and evolutionary trees were constructed by maximum likelihood method with the help of Mega5 software (http://www.megasoftware.net/).

The Blastp program was also used to search for putative nucleosome–binding regions in other species with similarity to the novel nucleosome binding region in SPBP and RAI1 (http://www.uniprot.org/). The alignment of the deduced amino acid sequences were carried out using ClustalW. 

### Cell imaging

6000 cells/well were seeded in eight-well cover glass slides (Nunc) and transfected with 100-200 ng of plasmid expression vectors using TransIT-LT1 (Mirus, MIR2300) reagent. One day post-transfection cells were analyzed by live-cell confocal microscopy and images were examined using Zeiss Axiovert 200 microscope with a 40X, 1.2W C-Apochroma objective, equipped with an LSM510-META confocal module. 

### Isolation of soluble mono- and di-nucleosomes and GST-nucleosome pull-down assay

Soluble mono- and di-nucleosomes from HeLa cells were prepared as described [20]. GST-nucleosome pull-down assay is described previously [20].

### GST pull-down assay

Expression and purification of GST, GST-fusion proteins and GST pull-down assay with *in vitro* translated ^35^S-labeled proteins were carried out as described [19].

## Results and Discussion

### Experimental validation of a zinc finger module N-terminal of the atypical PHD domain of SPBP

Alignment of the putative ePHD/ADD domains of SPBP and RAI1 with structurally known ePHD/ADD domains, suggests that an approximately 150 amino acid region N-terminal to the atypical PHD module may form a zinc finger structure similar to the ATRX/DNMT3 ePHD/ADD domains [20]. However, SPBP and RAI1 contain an extraordinary long loop region between the putative zinc ligands 2 and 3. The loop consists of more than 100 amino acids, and hence may form into a structure restricting the formation of a GATA-1 like zinc finger as reported for ATRX [31] and DNMT3 [32]. To experimentally evaluate the predicted model of SPBP/RAI1 ePHD/ADD domain (Figure 1B), we first experimentally validated interaction between the F-boxes of SPBP/RAI1, containing zinc ligands 1 and 2, and the C-terminal part of the SPBP/RAI1 ePHD/ADD domains starting from zinc ligand 3 (ePHD3-12). Fragments containing the F box region of SPBP and RAI1 were fused in frame with the GAL4 activation domain (GAL4-AD), and tested against different ePHD domain constructs of RAI1 and SPBP, fused to the GAL4 DNA binding domain (GAL4-DBD), in the yeast two-hybrid interaction system. Interestingly, the F boxes of both SPBP and RAI1 associated with the truncated ePHD domain (ePHD3-12) of both proteins (Figure 1C), but not with the ePHD/ADD domains (ePHD/ADD) of the trithorax family proteins; the histone methyltransferases mixed-lineage leukemia genes MLL1, MLL2 and MLL3 (Figure 1C). In addition, they also failed to interact with the complete ePHD domains (ePHD1-12) of SPBP and RAI1 in this assay system (Figure 1C and data not shown). The cross-reaction between the F-boxes of SPBP and RAI1 with their ePHD(3-12) are not surprising, since approximately 45% of the amino acids, including the two cysteine residues that are proposed to function as zinc-ligands, are conserved between their F boxes. 

 Since the complete ePHD/ADD domain (ePHD/ADD) of SPBP failed to interact with the F box in the two-hybrid system, we asked if such an interaction could be observed if we disrupted the putative zinc finger structure formed by ligands 1-4, by mutating ligands 1 and 2. The idea being that in the yeast two-hybrid system, the intramolecular binding between the F box and the core PHD zinc finger would prevent any intermolecular interactions to take place. Thus, by mutating zinc-ligands 1 and 2, the interactions surface on the core PHD would be available for interactions in trans. As seen in Figure 1D, mutation of both putative zinc ligands (1 and 2) in the F box, or only ligand 2 to alanine(s) enabled interactions in trans in the two-hybrid system. As a control, we mutated the cysteine residue between ligands 1 and 2, which is not thought to be a zinc-ligand. This mutation did not enable an interaction in trans. 

 Taken together, the results presented above indicate a binding mode where the F box in one molecule interacts with the ePHD domain in the other. However, the assays used measure intermolecular- and not intramolecular interactions. We suggest that within the ePHD/ADD domains of both SPBP and RAI1 the F box may bind to part of the ePHD3-12 region in the same molecule. To determine whether the PHD module, containing zinc-ligands 5 to 12 of the SPBP ePHD domain, displays an interaction surface used by the F box, the PHD of SPBP was fused to GAL4-DBD, generating GAL4-DBD-SPBP (ePHD5-12). Subsequently, this construct was tested for binding to the F box of SPBP in the yeast two-hybrid system. Interestingly, the SPBP PHD module associated with the F box, showing that an interaction surface sufficient for binding the F box residues is present in the PHD module (Figure 1D). To conclude, our results are compatible with the model shown in Figure 1B where the F box region interacts with the PHD module. This, together with the sequence homology between SPBP ePHD/ADD and other ePHD/ADD domains, suggest that SPBP ePHD/ADD share the cross-braced topology of zinc-binding interactions demonstrated for the ePHD/ADD domains of ATRX and DNMT3 [31,32].

### Both SPBP and RAI1 are present in vertebrates, while only one gene is found in insects and lancelet

**Figure 2:**
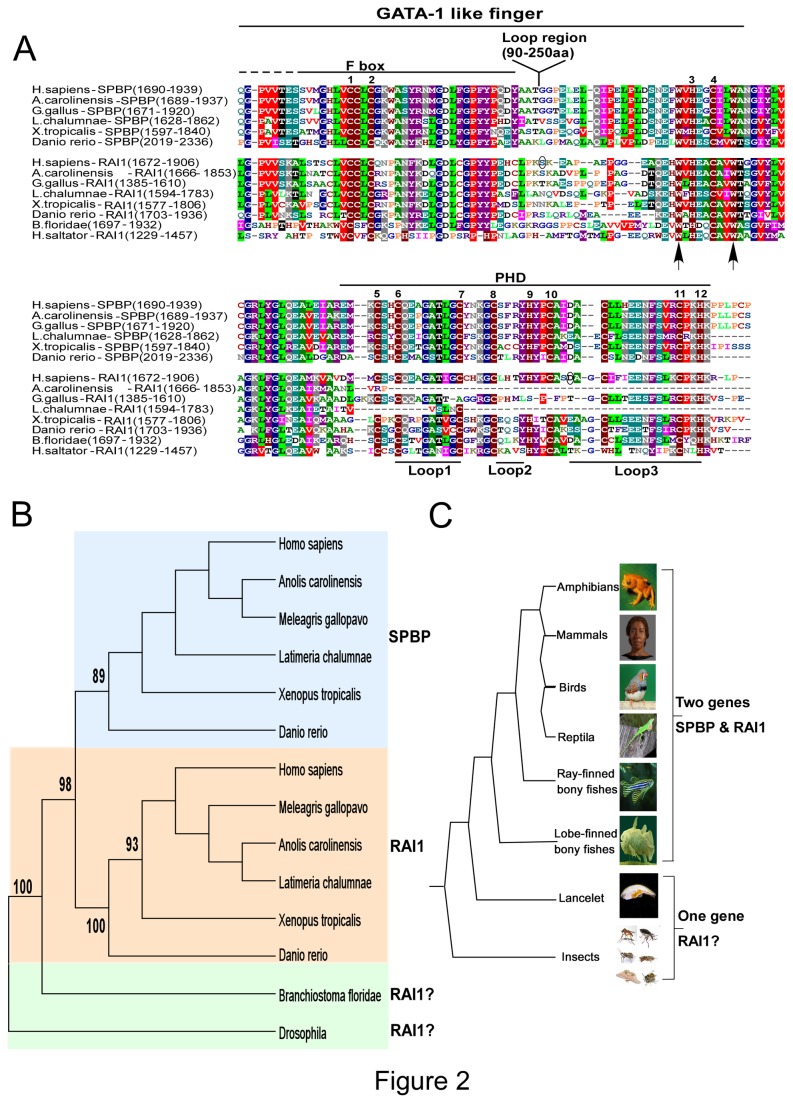
The ePHD/ADD domains of SPBP and RAI1 are conserved in evolution. (A) Alignment of the putative ePHD/ADD domain of SPBP and RAI1 in mammalia, reptiles, birds, amphibians, fishes and insect. Cysteine and histidine residues that may serve as zinc-ligands are indicated by numbers above the alignment. The long loop region between zinc ligands 2 and 3 in all species is excluded from the alignment, but indicated above. The regions encompassing the putative GATA-1 like finger and the PHD finger are indicated above, while the Loop1, Loop2 and Loop3 indicated below are loop structures generally found in PHD fingers. The conserved Tryptophan units used to select blasted sequences are indicated by arrows, while amino acids probably involved in Smith-Magenis syndrome are encircled. Two of the RAI1 sequences (A.carolinensis and L.chalumnae) are truncated in the C-terminal end probably due to sequencing errors. The threshold for shading in manually refined Clustal W sequence alignment was set to 50% using BLOSUM 62 scoring matrix. (B) Phylogenetic tree of the ePHD/ADD domains of SPBP and RAI1 proteins. The phylogenetic tree was constructed by Mega5 software using the maximum likelihood method. Numbers in the branch represents the bootstrap values. Background color coding is used to represent species which possess SPBP (blue), RAI1 (pink) and uncharacterized SPBP/RAI1 like proteins (green). The phylogenetic tree is based on selected protein sequences from representative vertebrate organisms (e.g. Homo sapiens represent Mammalia). (C) The evolutionary tree of the ePHD/ADD domains of SPBP and RAI1 proteins. The alignment of all sequences is shown in Supporting information, Figure S1 and the protein sequence accession numbers are provided in the Supporting information, Table S3.

The PHD finger represents one of the most abundant modules present in nuclear and chromatin binding proteins [recently reviewed in 33,34]. This structurally conserved motif consists of a two-strand antiparallel β-sheet often followed by a C-terminal α-helix, stabilized by two zinc atoms contacted by Cys4-His-Cys3 ligands forming a cross-branch topology (Figure 1B) [35]. PHD fingers are generally accepted as “readers” of the histone code, with ability to read the N-terminal tail of histone H3, even some PHD fingers are found not to bind histones or nucleosomes. Based on their sequence homology and structural plasticity, the PHD fingers may be divided into five subgroups each “reading” specific sets of histone marks [33,34]. PHD fingers are frequently paired with other “reader” modules, the PHD finger itself, and the GATA-1 finger that may both be a DNA binding module or a “reader” module [6], or with other DNA binding modules such as the AT-hook motif. Above and previously, we show that the PHD fingers of SPBP and RAI1 is closely linked to an N-terminal zinc finger structure with similarity to the ePHD/ADD fingers found in eight other groups of chromatin binding proteins [20]. However, since the extra zinc fingers of SPBP and RAI1 contain an extraordinary long loop between zinc ligands 2 and 3, they constitute a specific subgroup of ePHD/ADD modules. This hallmark of SPBP and RAI1 was used as criteria in BLAST search to retrieve putative SPBP and RAI1 sequences in genomes beside humans. In addition, selection was based on protein size and two conserved tryptophan residues, one two positions N-terminal to zinc-ligand 3 and one three positions C-terminal to zinc-ligand 4, in the GATA-1 like finger (Figure 2A). This approach yielded 64 SPBP and RAI1-like sequences in vertebrates spanning from bony fishes to mammals, in addition to 17 SPBP/RAI1-like sequence in lancelets and insects (Figure 2B/C). No SPBP or RAI1 like sequences were identified in fungi and plants, suggesting that SPBP/RAI1 like genes have occurred in the common ancestor of vertebrate and insects, and branched out in evolution. In figure 2, a representative selection of SPBP and RAI1 homologues and their distribution in a phylogenetic tree are presented. Both SPBP and RAI1proteins are found in vertebrate species like Western clawed frog (Amphibia), different mammals, birds, reptiles, zebra fish (Actinopterygii), and West Indian Ocean coelacanth (Sarcopterygii). A putative uncharacterized protein in invertebrate species such as Florida lancelet (Leptocardii) shows 38% sequence similarity to human RAI1 protein and various insect groups such as Drosophila, Mosquito and Ants species also show varying similarities with RAI1. From the phylogenetic and evolutionary trees we speculate that the RAI1 gene is the ancestral gene. A gene duplication event [36] may have taken place early in the vertebrate evolution, just after branching from insects, giving rise to SPBP. Alignment of the dataset from the retrieved SPBP and RAI1 ePHD/ADD domains resulted in a region with a length of approximately 150 amino acids (Supporting information, Figure S1), after removing the loop region between zinc ligand 2 and 3. Phylogenetic and evolutionary trees were constructed using the maximum likelihood method revealing two well supported clusters corresponding to SPBP and RAI1 in vertebrates (Figure 2B). The SPBP ePHD/ADD domains from zebrafish to human are highly conserved, with an overall sequence identity of 48%. Similarly, conservation of the RAI1 ePHD/ADD sequences are 33%. Interestingly, the conservation of all RAI1 and SPBP ePHD/ADD domains are high, especially the residues constituting the GATA-1 like finger (Figure 2A). Structural and biochemical analysis of the GATA-1 finger in the ATRX ePHD/ADD domain has revealed that it is a “reader” module, recognizing H3K9me2/3. The residues involved (Tyr203, Ile209, and Ala224) are localized in the region between zinc-ligand 4 of the GATA-1 finger, and zinc-ligand 3 in the ATRX PHD finger, which forms a polar K9me3 reader pocket [37]. This region between zinc ligands 4 and 6 is highly conserved between the SPBP and RAI1 proteins, containing several conserved hydrophobic residues (gly, val, leu, ala, tyr, trp), in addition to conserved basic and acidic residues, similarly as ATRX. The positions corresponding to the three amino acids involved in H3K9me3 recognition (Tyr203, Ile209, and Ala224) in ATRX, are conserved and occupied by amino acids with similar properties (Trp, Leu, Gln respectively) in SPBP and RAI1. These similarities suggest that the GATA-1 like fingers of SPBP and RAI1 may constitute histone reader modules, recognizing specific histone marks such as the ATRX GATA-1 like finger. This hypothesis, suggesting the GATA-1 like finger to be a histone binding module, is further supported by the structural analysis of the PHD finger of UHRF1 [38]. Here the PHD finger including an additional N-terminal zinc-finger motif together forms a large surface cavity that interacts with the N-terminal tail of Histone H3. Notably, the GATA-1 like fingers of SPBP and RAI1 exhibit this long loop region between zinc ligand 2 and 3. The loop region is poorly conserved and not included in the phylogenetic data sets. Comparison with the structure of ATRX suggests that this long loop region may interact with the long C-terminal α-helix often present in ePHD/ADD domains [6]. In SPBP, several serine and threonine residues within the long loop region is reported to be phosphorylated (www.phosphosite.org). Interestingly, a missense mutation of serine 1808 in the long loop of RAI1 (S1808N) is connected to Smith-Magenis Syndrome [39] indicating that the long loop is important for proper function of RAI1. Furthermore, phosphorylation of a residue in a loop region between the ePHD/ADD finger and the Tudor domain of UHRF1, can modulate the relative position of the reader modules and thereby alter its histone binding mode [9]. This indicates that the long loop regions between zinc ligand 2 and 3 in the GATA-1 like zinc finger may have regulatory roles modulating the SPBP/RAI1-nucleosome interaction. 

 The PHD fingers of SPBP and RAI1 are atypical, displaying “Cys4HisCys2His” fingers lacking the conserved tryptophan residue characteristic of the conventional PHD finger [20]. Figure 2A reveals that the two loop regions, Loop1 and Loop3, in the PHD are highly conserved. Loop1 is glycine rich containing glutamate and a conserved Threonine residue. Hence, this seems to be an acidic region, and phosphorylation of the threonine residue will make Loop1 even more acidic. Specific tyrosine and methionine residues in Loop1 are involved in recognizing H3K4me2/3 marks in certain PHD subgroups [34]. These residues are not present in the RAI1/SPBP PHDs, confirming our previous finding that the SPBP PHD does not recognize the H3K4me2/3 marks. For both proteins, Loop3 is rich in acidic residues, in addition to a conserved serine residue and a bulge hydrophobic Phenylalanine residue. Interestingly, mutation of one of the charged amino acids in Loop3 of RAI1 (D1885N) may be involved in familial Smith-Magenis syndrome [40]. Loop3 in the PHDs of PYGO1 and MLL proteins, are involved in protein-protein interactions with non-histone partners. We have previously reported the ePHD/ADD domain of SPBP to be important for specific protein-protein interactions [19]. Hence, Loop3 in the PHD of SPBP and RAI1 may constitute a negatively charged interaction surface important for recruitment of proteins to specific chromatin sites.

### The novel chromatin binding region including the adjacent AT-hook motif is conserved in evolution

**Figure 3:**
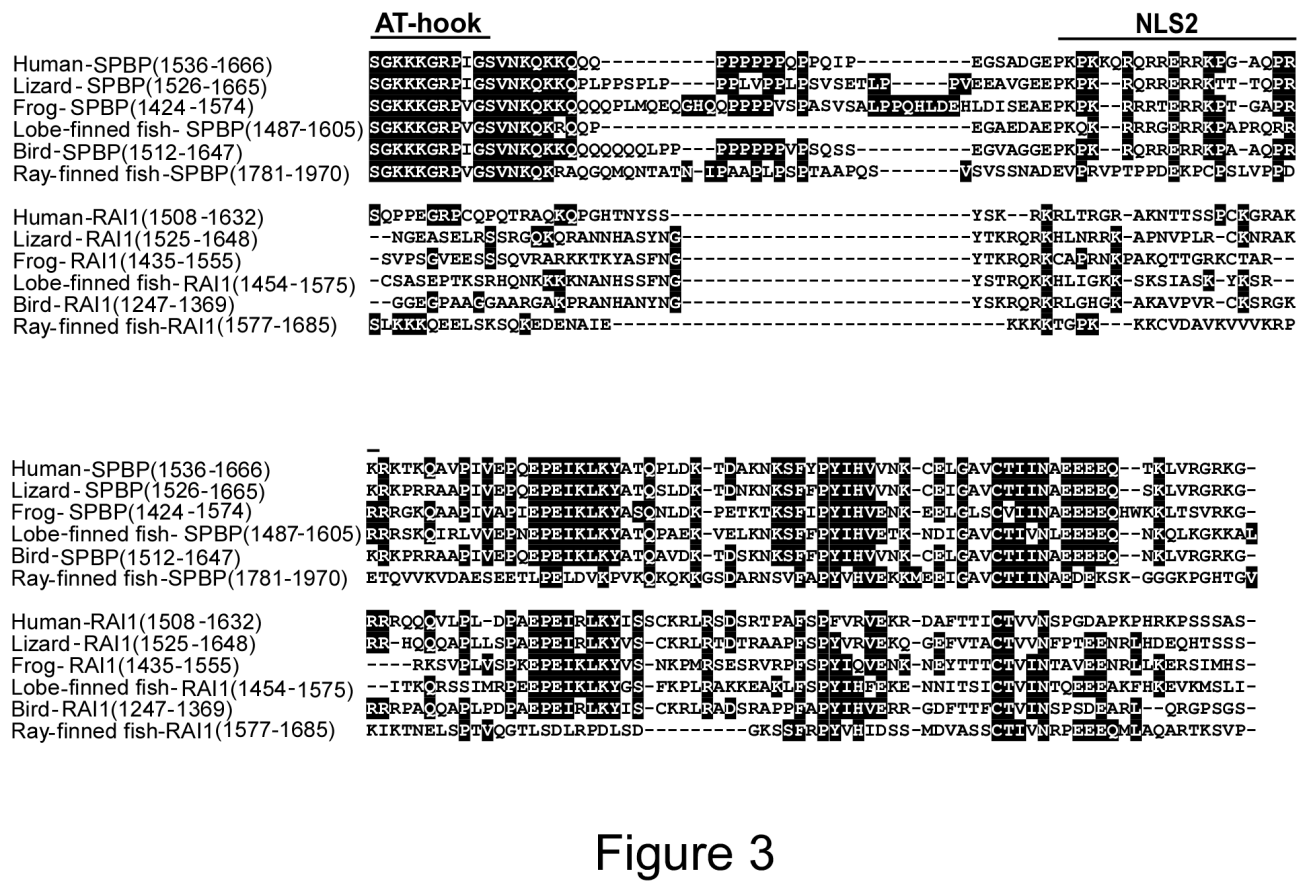
The novel nucleosome binding region of SPBP is conserved. Alignment of the novel nucleosome binding region of SPBP, and the corresponding region of RAI1, in human, lizard, frog, fishes and bird species. In ray-finned fish a 42 amino acid insertion (position 1923-1965) is omitted from the alignment. Position of AT-hook motif and a NLS signal is indicated above. The protein sequence accession numbers are provided in the Supporting information, Table S4, respectively.

Previously we have identified a novel nucleosome binding region adjacent to the DNA binding domain with an AT-hook motif in human SPBP [20]. Sequence alignments predicted a similar region in human RAI1, however a complete AT hook motif could not be detected and a RAI1 DNA binding region has not been described in the literature. To investigate whether the nucleosome-binding region following an AT-hook motif is a conserved feature of the SPBP and RAI1 proteins, blast searches and manual inspection of the corresponding sequences were carried out. Alignment of the dataset from the novel nucleosome binding regions resulted in a region of approximately 130 amino acids (Figure 3A). Alignments and phylogenetic studies divided the sequences into two clusters corresponding to SPBP-like proteins and RAI1 like proteins. The average sequence identity between the novel nucleosome binding regions in SPBP proteins was 65%, while the identity in the RAI1 cluster was 33%. Interestingly, the AT-hook motif is completely conserved in the SPBP proteins, indicating that DNA binding activity is an important feature (Figure 3A). In RAI1 however, no AT-hook motif is present and the conservation of this region is low. Only in mammalian RAI proteins, the core GRP motif of the AT-hook is present. If RAI1 is the ancient protein, this GRP motif may be a remnant of a DNA binding activity lost in evolution. The C-terminal part of the novel nucleosome binding region is strongly conserved between SPBP and RAI1 in all species. This region exhibit the E-box (Figure 1A), previously reported to be similar between SPBP and RAI1 in humans [21]. 

### The putative nucleosome binding region of RAI1 displays nucleosome and histone binding activity in vitro

**Figure 4:**
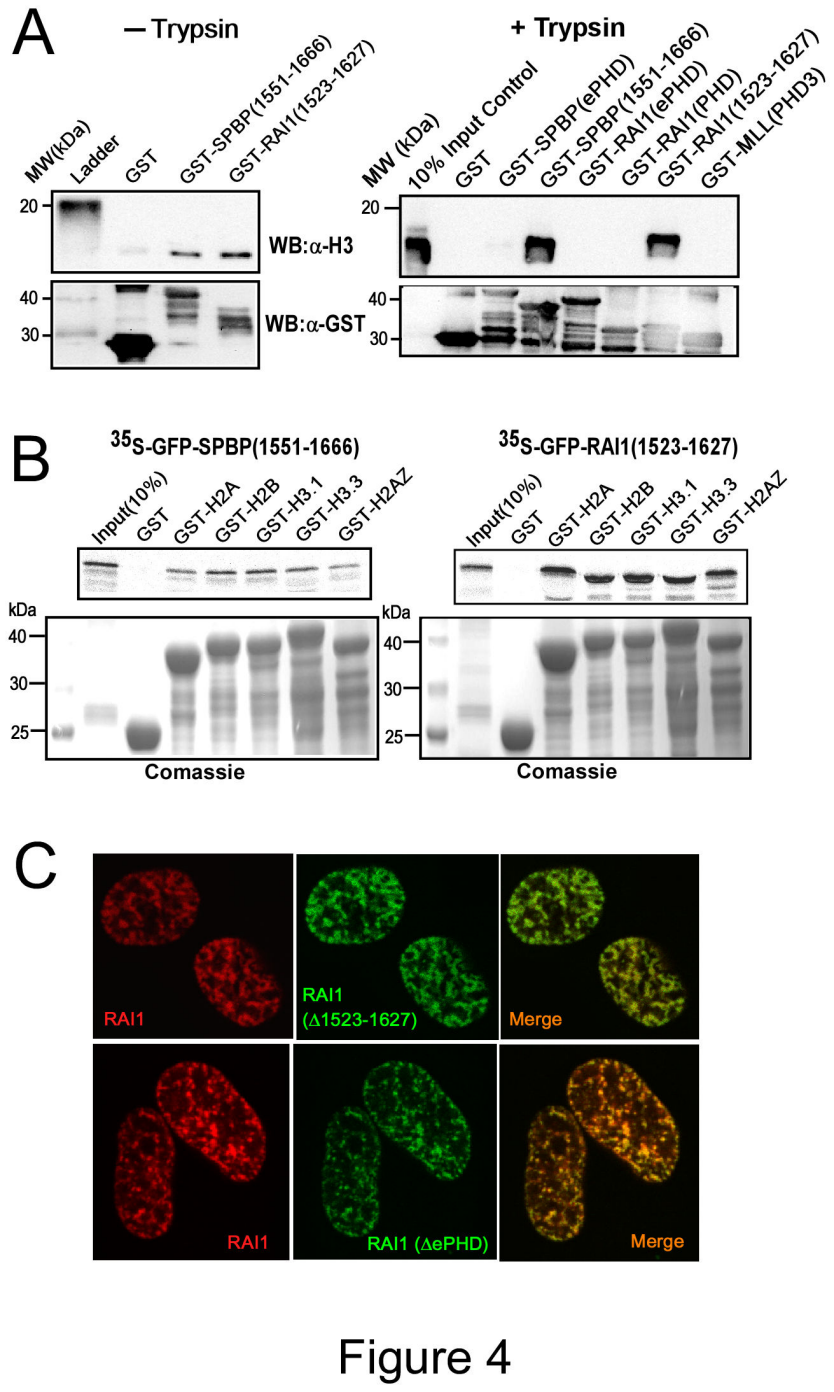
The RAI1 region 1523-1627 has the ability to bind nucleosomes and histones *in* *vitro*. (**A**) The region of RAI1 with homology to the novel nucleosome binding region in SPBP binds HeLa nucleosomes independent of the histone tails. Intact nucleosomes isolated from HeLa cells were incubated with GST, GST-SPBP (1551-1666), and GST-RAI1(1523-1627) (left panel), and partial trypsin digested nucleosomes were incubated with GST, GST-GST-SPBP(ePHD), GST-SPBP(1551-1666), GST-RAI1(ePHD), GST-RAI1(PHD), GST-RAI1(1523-1627) and negative control GST-MLL(PHD3) (right panel) as indicated. Bound proteins were subjected to Western blotting using anti–histone H3 antibody and anti-GST antibody. (**B**) The novel nucleosome-binding region 1551-1666 of SPBP and 1523-1627 of RAI1 interacts with histones H2 and H3 variants. Labeled GFP-SPBP (1551-1666) and GFP-RAI1(1523-1627) were incubated with GST, GST-H2A, H2B, H3.1, H3.3 and H2AZ expressed and purified from *E. coli*. Interacting proteins were subjected to SDS-PAGE and visualized using a PhosphorImager. (**C**) Deletion of the novel chromatin binding region of RAI1 (1523-1627), or the ePHD domain of RAI1, has no significant impact on RAI1 distribution in the nucleus of HeLa cells. HeLa cells were transiently co-transfected with plasmid expressing mCherry-RAI1 and EGFP-RAI1 (Δ1523-1627) (upper panels), or mCherry-RAI1 and EGFP-RAI1(ΔePHD)(lower panels).

Based on the conservation of the region encompassing the novel SPBP nucleosome binding region; we wanted to determine whether this region of RAI1 has the ability to bind nucleosomes and histones. The corresponding region of RAI1 was expressed as a GST-fusion protein and exposed to nucleosomes isolated from HeLa cells in a GST-nucleosome binding assay. The results presented in Figure 4A show that RAI1 (1523-1627) binds to HeLa nucleosomes *in vitro* equally well as SPBP (1551-1666). This binding was not dependent on the histone tails, since partial Trypsin digestion of the nucleosomes did not reduce binding (Figure 4A, right panel). The RAI(ePHD) and RAI(PHD), on the other hand, did not bind to Trypsin treated nucleosomes, indicating that the nucleosome binding activity of the RAI(ePHD) is histone tail dependent, as previously found for the SPBP(ePHD) domain [20]. Furthermore, GST-pulldown assays using various Histone H3 and Histone H2 variants, showed strong interaction between RAI1(1523-1627) and histones (Figure 4b). This is in line with our previous FRAP analysis of the RAI1 mobility in HeLa cells, which indicated strong association to nuclear structures [20]. Deletion of the region encompassing the novel nucleosome binding region in SPBP resulted in redistribution into round nuclear dots instead of the characteristic speckled distribution due to enrichment on chromatin [20]. To determine whether this corresponding nucleosome binding region of RAI1 was important for its nuclear distribution, a deletion construct (Δ1523-1627) of RAI1 fused to EGFP was transfected into HeLa cells and its localization analyzed by confocal microscopy (Figure 4C). The nuclear distribution of RAI1(Δ1523-1627) was similar to full length RAI1 in all cells, indicating that also other regions of RAI1 are important for its localization to chromatin. A strong candidate here is the ePHD/ADD domain. However, a deletion construct expressing RAI1 without the ePHD/ADD domain displayed similar localization as full length RAI1. This strongly indicate that both the ePHD/ADD and the 1523-1627 region of RAI1 contributes to its nuclear distribution pattern. The presence of several regions in a protein that contribute to chromatin localization is shown for other proteins [6]. To summarize, the novel nucleosome binding region previously identified in SPBP is evolutionary conserved and present also in RAI1 proteins. However, in contrast to SPBP, RAI1 proteins are generally lacking the adjacent AT-hook motif, and the novel nucleosome binding region does not contribute alone to the enrichment of RAI1 on chromatin.

## Conclusion

Here we have shown that SPBP and RAI1 are evolutionary conserved nuclear proteins containing several motifs for chromatin interactions. They exist as two different genes in vertebrates, while only one gene was identified in insects and lancelet. Two conserved regions for chromatin interaction were identified in RAI1, a novel nucleosome binding region located between amino acids 1523 and 1627, and the C-terminal atypical PHD finger connected to a GATA-1 like zinc finger structure (ePHD/ADD domain). SPBP on the other hand, seems to exhibit several conserved regions involved in DNA and chromatin binding; the novel nucleosome binding region, the AT-hook motif, and the ePHD/ADD domain. Interestingly, SPBP also contains a putative HP1 binding region (PxVxL) at position 1329 (PAVTL). All these regions are localized in the C-terminal half of the protein, while most of the N-terminal part has been identified as a transcriptional activation domain, suggesting that it has strong capability to interact with other proteins involved in transcription. Interaction of SPBP with the ligand activated transcription factors Androgen receptor and Estrogen receptor is known [23,24]. Interestingly, a yeast two-hybrid screen acknowledged the RNA polymerase II subunit Rpb7, the RNA binding proteins hnRNPH/F, the SWI/SNF complex protein ARID1B and the chromatin modulator G9a as putative interaction partners of SPBP (our unpublished results). This suggests that SPBP and RAI1 may act as scaffold proteins, mediating interaction between specific sites on chromatin with chromatin modulation complexes, transcriptional regulators and the general transcription machinery simultaneously. Hence, SPBP and RAI1 may have an important role in integration of signaling pathways and regulation of gene transcription. Notably, the interaction between SPBP/RAI1 and chromatin seems to be strictly regulated, since several sites within the ePHD/ADD domain and the novel nucleosome binding domain is targeted by post-translational modifications [www.phososite.org, [20]]. This putative ability to mediate regulated cross talk between the “histone code” and diverse cellular pathways that the non-histone partners participate in, seems to be connected to several other multidomain proteins containing chromatin “reader” domains [6-17,33,41]. 

## Supporting Information

Figure S1 **Alignment of the ePHD/ADD domain of SPBP, RAI1 and SPBP/RAI1 like proteins in different species.**
(PDF)

Table S1 **cDNA constructs made in this study.**
(DOCX)

Table S2 **Primers used in this study.**
(DOCX)

Table S3 **The ePHD/ADD domain of SPBP and RAI1 in different species.**
(DOCX)

Table S4 **Novel nucleosome-binding region of SPBP and RAI1 in different species.**
(DOCX)

## References

[1] Kwon CS, Wagner D (2007) Unwinding chromatin for development and growth: a few genes at a time. Trends Genet 23: 403-412. doi:10.1016/j.tig.2007.05.010. PubMed: 17566593. 17566593

[2] Daniel JA, Grant PA (2007) Multi-tasking on chromatin with the SAGA coactivator complexes. Mutat Res 618: 135-148. doi:10.1016/j.mrfmmm.2006.09.008. PubMed: 17337012. 17337012 PMC1892243

[3] Spiegelman BM, Heinrich R (2004) Biological control through regulated transcriptional coactivators. Cell 119: 157-167. doi:10.1016/j.cell.2004.09.037. PubMed: 15479634. 15479634

[4] Thorne JL, Campbell MJ, Turner BM (2009) Transcription factors, chromatin and cancer. Int J Biochem Cell Biol 41: 164-175. doi:10.1016/j.biocel.2008.08.029. PubMed: 18804550. 18804550

[5] Taverna SD, Li H, Ruthenburg AJ, Allis CD, Patel DJ (2007) How chromatin-binding modules interpret histone modifications: lessons from professional pocket pickers. Nat Struct Mol Biol 14: 1025-1040. doi:10.1038/nsmb1338. PubMed: 17984965. 17984965 PMC4691843

[6] Eustermann S, Yang JC, Law MJ, Amos R, Chapman LM et al. (2011) Combinatorial readout of histone H3 modifications specifies localization of ATRX to heterochromatin. Nat Struct Mol Biol 18: 777-782. doi:10.1038/nsmb.2070. PubMed: 21666677. 21666677

[7] Zeng L, Zhang Q, Li S, Plotnikov AN, Walsh MJ et al. (2010) Mechanism and regulation of acetylated histone binding by the tandem PHD finger of DPF3b. Nature 466: 258-262. doi:10.1038/nature09139. PubMed: 20613843. 20613843 PMC2901902

[8] Aravind L, Iyer LM (2012) The HARE-HTH and associated domains: novel modules in the coordination of epigenetic DNA and protein modifications. Cell Cycle 11: 119-131. doi:10.4161/cc.11.1.18475. PubMed: 22186017. 22186017 PMC3272235

[9] Arita K, Isogai S, Oda T, Unoki M, Sugita K et al. (2012) Recognition of modification status on a histone H3 tail by linked histone reader modules of the epigenetic regulator UHRF1. Proc Natl Acad Sci U S A 109: 12950-12955. doi:10.1073/pnas.1203701109. PubMed: 22837395. 22837395 PMC3420164

[10] Callebaut I, Mornon JP (2012) The PWAPA cassette: Intimate association of a PHD-like finger and a winged-helix domain in proteins included in histone-modifying complexes. Biochimie 94: 2006-2012. doi:10.1016/j.biochi.2012.05.025. PubMed: 22664638. 22664638

[11] Morra R, Lee BM, Shaw H, Tuma R, Mancini EJ (2012) Concerted action of the PHD, chromo and motor domains regulates the human chromatin remodelling ATPase CHD4. FEBS Lett 586: 2513-2521. PubMed: 22749909. 22749909 10.1016/j.febslet.2012.06.017 PMC3476528

[12] Watson AA, Mahajan P, Mertens HD, Deery MJ, Zhang W et al. (2012) The PHD and Chromo Domains Regulate the ATPase Activity of the Human Chromatin Remodeler CHD4. J Mol Biol 422: 3-17. doi:10.1016/j.jmb.2012.04.031. PubMed: 22575888. 22575888 PMC3437443

[13] Kumar GS, Chang W, Xie T, Patel A, Zhang Y et al. (2012) Sequence Requirements for Combinatorial Recognition of Histone H3 by the MRG15 and Pf1 Subunits of the Rpd3S/Sin3S Corepressor Complex. J Mol Biol 422: 519-531. doi:10.1016/j.jmb.2012.06.013. PubMed: 22728643. 22728643 PMC3428507

[14] Xie S, Jakoncic J, Qian C (2012) UHRF1 double tudor domain and the adjacent PHD finger act together to recognize K9me3-containing histone H3 tail. J Mol Biol 415: 318-328. doi:10.1016/j.jmb.2011.11.012. PubMed: 22100450. 22100450

[15] Oliver SS, Musselman CA, Srinivasan R, Svaren JP, Kutateladze TG et al. (2012) Multivalent Recognition of Histone Tails by the PHD Fingers of CHD5. Biochemistry 51: 6534-6544. doi:10.1021/bi3006972. PubMed: 22834704. 22834704 PMC3518043

[16] Qiu Y, Liu L, Zhao C, Han C, Li F et al. (2012) Combinatorial readout of unmodified H3R2 and acetylated H3K14 by the tandem PHD finger of MOZ reveals a regulatory mechanism for HOXA9 transcription. Genes Dev 26: 1376-1391. doi:10.1101/gad.188359.112. PubMed: 22713874. 22713874 PMC3387664

[17] Dhayalan A, Tamas R, Bock I, Tattermusch A, Dimitrova E et al. (2011) The ATRX-ADD domain binds to H3 tail peptides and reads the combined methylation state of K4 and K9. Hum Mol Genet 20: 2195-2203. doi:10.1093/hmg/ddr107. PubMed: 21421568. 21421568 PMC3090196

[18] Bi W, Ohyama T, Nakamura H, Yan J, Visvanathan J et al. (2005) Inactivation of Rai1 in mice recapitulates phenotypes observed in chromosome engineered mouse models for Smith-Magenis syndrome. Hum Mol Genet 14: 983-995. doi:10.1093/hmg/ddi085. PubMed: 15746153. 15746153

[19] Sjottem E, Rekdal C, Svineng G, Johnsen SS, Klenow H, et al (2007) The ePHD protein SPBP interacts with TopBP1 and together they co-operate to stimulate Ets1-mediated transcription. Nucleic Acids Res 35: 6648-6662. 17913746 10.1093/nar/gkm739 PMC2095823

[20] Darvekar S, Johnsen SS, Eriksen AB, Johansen T, Sjøttem E (2012) Identification of two independent nucleosome-binding domains in the transcriptional co-activator SPBP. Biochem J 442: 65-75. doi:10.1042/BJ20111230. PubMed: 22081970. 22081970

[21] Rekdal C, Sjøttem E, Johansen T (2000) The nuclear factor SPBP contains different functional domains and stimulates the activity of various transcriptional activators. J Biol Chem 275: 40288-40300. doi:10.1074/jbc.M006978200. PubMed: 10995766. 10995766

[22] Sanz L, Moscat J, Diaz-Meco MT (1995) Molecular characterization of a novel transcription factor that controls stromelysin expression. Mol Cell Biol 15: 3164-3170. PubMed: 7760812. 7760812 10.1128/mcb.15.6.3164 PMC230548

[23] Elvenes J, Thomassen EI, Johnsen SS, Kaino K, Sjøttem E et al. (2011) Pax6 Represses Androgen Receptor-Mediated Transactivation by Inhibiting Recruitment of the Coactivator SPBP. PLOS ONE 6: e24659. doi:10.1371/journal.pone.0024659. PubMed: 21935435. 21935435 PMC3174178

[24] Gburcik V, Bot N, Maggiolini M, Picard D (2005) SPBP is a phosphoserine-specific repressor of estrogen receptor alpha. Mol Cell Biol 25: 3421-3430. doi:10.1128/MCB.25.9.3421-3430.2005. PubMed: 15831449. 15831449 PMC1084313

[25] Slager RE, Newton TL, Vlangos CN, Finucane B, Elsea SH (2003) Mutations in RAI1 associated with Smith-Magenis syndrome. Nat Genet 33: 466-468. doi:10.1038/ng1126. PubMed: 12652298. 12652298

[26] Potocki L, Bi W, Treadwell-Deering D, Carvalho CM, Eifert A et al. (2007) Characterization of Potocki-Lupski syndrome (dup(17)(p11.2p11.2)) and delineation of a dosage-sensitive critical interval that can convey an autism phenotype. Am J Hum Genet 80: 633-649. doi:10.1086/512864. PubMed: 17357070. 17357070 PMC1852712

[27] Toulouse A, Rochefort D, Roussel J, Joober R, Rouleau GA (2003) Molecular cloning and characterization of human RAI1, a gene associated with schizophrenia. Genomics 82: 162-171. doi:10.1016/S0888-7543(03)00101-0. PubMed: 12837267. 12837267

[28] van der Zwaag B, Franke L, Poot M, Hochstenbach R, Spierenburg HA et al. (2009) Gene-network analysis identifies susceptibility genes related to glycobiology in autism. PLOS ONE 4: e5324. doi:10.1371/journal.pone.0005324. PubMed: 19492091. 19492091 PMC2683930

[29] Hayes S, Turecki G, Brisebois K, Lopes-Cendes I, Gaspar C et al. (2000) CAG repeat length in RAI1 is associated with age at onset variability in spinocerebellar ataxia type 2 (SCA2). Hum Mol Genet 9: 1753-1758. doi:10.1093/hmg/9.12.1753. PubMed: 10915763. 10915763

[30] Lamark T, Perander M, Outzen H, Kristiansen K, Øvervatn A et al. (2003) Interaction codes within the family of mammalian Phox and Bem1p domain-containing proteins. J Biol Chem 278: 34568-34581. doi:10.1074/jbc.M303221200. PubMed: 12813044. 12813044

[31] Argentaro A, Yang JC, Chapman L, Kowalczyk MS, Gibbons RJ et al. (2007) Structural consequences of disease-causing mutations in the ATRX-DNMT3-DNMT3L (ADD) domain of the chromatin-associated protein ATRX. Proc Natl Acad Sci U S A 104: 11939-11944. doi:10.1073/pnas.0704057104. PubMed: 17609377. 17609377 PMC1924575

[32] Otani J, Nankumo T, Arita K, Inamoto S, Ariyoshi M et al. (2009) Structural basis for recognition of H3K4 methylation status by the DNA methyltransferase 3A ATRX-DNMT3-DNMT3L domain. EMBO Rep 10: 1235-1241. doi:10.1038/embor.2009.218. PubMed: 19834512. 19834512 PMC2775176

[33] Sanchez R, Zhou MM (2011) The PHD finger: a versatile epigenome reader. Trends Biochem Sci 36: 364-372. PubMed: 21514168. 21514168 10.1016/j.tibs.2011.03.005 PMC3130114

[34] Li Y, Li H (2012) Many keys to push: diversifying the 'readership' of plant homeodomain fingers. Acta Biochim Biophys Sinica 44: 28-39. doi:10.1093/abbs/gmr117. PubMed: 22194011. 22194011

[35] Kwan AH, Gell DA, Verger A, Crossley M, Matthews JM et al. (2003) Engineering a protein scaffold from a PHD finger. Structure 11: 803-813. doi:10.1016/S0969-2126(03)00122-9. PubMed: 12842043. 12842043

[36] Bi W, Saifi GM, Shaw CJ, Walz K, Fonseca P et al. (2004) Mutations of RAI1, a PHD-containing protein, in nondeletion patients with Smith-Magenis syndrome. Hum Genet 115: 515-524. doi:10.1007/s00439-004-1187-6. PubMed: 15565467. 15565467

[37] Iwase S, Xiang B, Ghosh S, Ren T, Lewis PW et al. (2011) ATRX ADD domain links an atypical histone methylation recognition mechanism to human mental-retardation syndrome. Nat Struct Mol Biol 18: 769-776. doi:10.1038/nsmb.2062. PubMed: 21666679. 21666679 PMC3130887

[38] Lallous N, Legrand P, McEwen AG, Ramón-Maiques S, Samama JP et al. (2011) The PHD finger of human UHRF1 reveals a new subgroup of unmethylated histone H3 tail readers. PLOS ONE 6: e27599. doi:10.1371/journal.pone.0027599. PubMed: 22096602. 22096602 PMC3214078

[39] Girirajan S, Elsas LJ 2nd, Devriendt K, Elsea SH (2005) RAI1 variations in Smith-Magenis syndrome patients without 17p11.2 deletions. J Med Genet 42: 820-828. doi:10.1136/jmg.2005.031211. PubMed: 15788730. 15788730 PMC1735950

[40] Vilboux T, Ciccone C, Blancato JK, Cox GF, Deshpande C et al. (2011) Molecular analysis of the Retinoic Acid Induced 1 gene (RAI1) in patients with suspected Smith-Magenis syndrome without the 17p11.2 deletion. PLOS ONE 6: e22861. doi:10.1371/journal.pone.0022861. PubMed: 21857958. 21857958 PMC3152558

[41] Migliori V, Mapelli M, Guccione E (2012) On WD40 proteins: Propelling our knowledge of transcriptional control? Epigenetics : official journal of the DNA Methylation Society 7: 815–22. PubMed: 22810296. 10.4161/epi.21140 PMC3427277 22810296

