# Supplementary material for: A Phylogenetic Study of SPBP and RAI1: Evolutionary Conservation of Chromatin Binding Modules
Source: PLoS One. 2013 Oct 18;8(10):e78907. doi: 10.1371/journal.pone.0078907 (PMC3799622; doi:10.1371/journal.pone.0078907)
Supplement: Figure S1 — Alignment of the ePHD/ADD domain of SPBP, RAI1 and SPBP/RAI1 like proteins in different species. (PDF) [file pone.0078907.s001.pdf]

F box

```
                                                 1  2
Q9UGU0(1690-1939)   QG---PVVTESSVMGHLVCCLCGKWASYRNMGDLFGPFYPQDYAAT--LP
G3QDX4(1690-1939)   QG---PVVTESSVMGHLVCCLCGKWASYRNMGDLFGPFYPQDYAAT--LP
H2QLT9(1692-1941)   QG---PVVTESSVMGHLVCCLCGKWASYRNMGDLFGPFYPQDYAAT--LP
H9FZ14(1690-1939)   QG---PVVTESSVMGHLVCCLCGKWASYRNMGDLFGPFYPQDYAAT--LP
G1S175(1688-1939)   QG---PVVTESSVMGHLVCCLCGKWASYRNMGDLFGPFYPQDYAAT--LP
H0XML0(1702-1951)   QG---PVVTESSVMGHLVCCLCGKWASYRNMGDLFGPFYPQDYAAT--LP
I3NC70(1718-1966)   QG---PVVTESSVMGHLVCCLCGKWASYRNMGDLFGPFYPQDYAAT--LP
F7CRS8(1718-1967)   QG---PVVTESSVMGHLVCCLCGKWASYRNMGDLFGPFYPQDYAAT--LP
E2RT08(1689-1938)   QG---PVVTESSVMGHLVCCLCGKWASYRNMGDLFGPFYPQDYAAT--LP
G1P5A9(1705-1953)   QG---PVVTESSVMGHLVCCLCGKWASYRNMGDLFGPFYPQDYAAT--LP
E1B8T3(1699-1947)   QG---PVVTESSVMGHLVCCLCGKWASYRNMGDLFGPFYPQDYAAT--LP
G3U8A2(1705-1954)   QG---PVVTESSVMGHLVCCLCGKWASYRNMGDLFGPFYPQDYAAT--LP
G1LSB5(1679-1927)   QG---PVVTESSVMGHLVCCLCGKWASYRNMGDLFGPFYPQDYAAT--LP
Q9EPQ8(1718-1962)   QG---PVVTESSVMGHLVCCLCGKWASYRNMGDLFGPFYPQDYAAT--LP
G1SYZ5(1632-1880)   QG---PVVTESSVMGHLVCCLCGKWASYRNMGDLFGPFYPQEYAAT--LP
H0VVM3(1651-1885)   QG---PVVTESSVMGHLVCCLCGKWASYRNMGDLFGPFYPQDYAAT--LP
F6Q9R3(1692-1946)   QG---PVVTESSVLGHLVCCLCGKWASYRNMGDLFGPFYPQDYVAT--LP
G3WIE0(1668-1874)   QG---PVVTESSVLGHLVCCLCGKWASYRNMGDLFGPFYPQDYVAT--LP
H0ZHA4(1664-1913)   QG---PVVTESSVLGHLVCCLCGKWASYRNMGDLFGPFYPQDYAAT--LP
E1BXI6(1671-1920)   QG---PVVTESSVLGHLVCCLCGKWASYRNMGDLFGPFYPQDYAAT--LP
G1NHL2(1672-1921)   QG---PVVTESSVLGHLVCCLCGKWASYRNMGDLFGPFYPQDYAAT--LP
G1KC34(1689-1937)   QG---PVVTESSVMGHLVCCLCGKWASYRNMGDLFGPFYPQDYAAT--LP
F7BZK2(1597-1840)   QG---PAVTESATMGHLVCCLCGKWANYRNLGDLFGPFYNQEYAST--LS
I3JNR0(1311-1549)   SG---PVITETNHSGRLLCCLCQKWANYKHLGDLYGPFYPAEYAAK--LP
D3ZG21(1722-1965)   QG---PVVTESSVMGHLVCCLCGKWASYRNMGDLFGPFYPQDYAAT--LP
G7PFP9(1725-1980)   QG---PVVTESSVMGHLVCCLCGKWASYRNMGDLFGPFYPQDYAAT--LP
H2P4M2(1692-1935)   QG---PVVTESSVMGHLVCCLCGKWASYRNMGDLFGPFYPQDYAAT--LP
H2ZXL0(1628-1862)   QG---PAVTESSVVGRLVCCLCGKWANYRSLGDLFGPYYPHDYIAT--LP
G3I0E6(721-964)     QG---PVVTESSVMGHLVCCLCGKWASYRNMGDLFGPFYPQDYAAT--LP
G5BPR9(1037-1303)   QG---PVVTESSVMGHLVCCLCGKWASYRNMGDLFGPFYPQDYAAT--LP
Q4SCG6(1995-2332)   VG---PVITETNHSGRLLCSLCQKWANYKNLGDLYGPFYPAEYAAR--LP
E7FE16(2019-2336)   PG---PVISETGHSGHLLCCLCQKWANYKHLGDLYGPFYPAEYAAK--LP
F1SRF7(1698-1893)   QG---PVVTESSVMGHLVCCLCGKWASYRNMGDLFGPFYPQDYAAT--LP
H9KVS1(1635-1821)   QG---PVVTESSVMGHLVCCLCGKWASYRNMGDLFGPFYPQDYAAT--LP
Q7Z5J4(1672-1906)   LG---PVVSKALSTSCLVCCLCQNPANFKDLGDLCGPYYPEHCLPKKKPK
H2QCD6(1667-1901)   LG---PVVSKALSTSCLVCCLCQNPANFKDLGDLCGPYYPEHCLPKKKPK
F7E488(1674-1909)   LG---PVVSKALSTSCLVCCLCQNPANFKDLGDLCGPYYPEHCLPKKKPK
H2NSX0(1678-1908)   LG---PVVSKALSTSCLVCCLCQNPANFKDLGDLCGPYYPEHCLPKK--K
G1RTB7(1668-1903)   LG---PVVSKALSTSCLVCCLCQNPANFKDLGDLCGPYYPEHCLPKKKPK
E1B9X1(1644-1885)   LG---PVVSKALSTSCLVCCLCQNPANFKDLGDLCGPYYPEHCLPKKKPK
D4A4Z4(1638-1887)   LG---PVVSKALSTSCLVCCLCQNPANFKDLGDLCGPYYPEHCLPKKKPK
Q61818(1640-1889)   LG---PVVSKALSTSCLVCCLCQNPANFKDLGDLCGPYYPEHCLPKKKPK
G1LF06(1637-1873)   LG---PVVSKALSTSCLVCCLCQNPANFKDLGDLCGPYYPEHCLPKKKPK
G3QJR9(1645-1879)   LG---PVVSKALSTSCLVCCLCQNPANFKDLGDLCGPYYPEHCLPKKKPK
G3TJ97(1586-1821)   LG---PVVSKALSTSCLVCCLCQNPANFKDLGDLCGPYYPEHCLPKKKPK
H0V1B2(1316-1545)   LG---PVVSKALSTSCLVCCLCQNPANFKDLGDLCGPYYPEHCLPKKKPK
I3N2I1(1670-1914)   LG---PVVSKALSTSCLVCCLCQNPANFKDLGDLCGPYYPEHCLPKKKPK
F7DZK3(1652-1884)   LG---PVVSKALSTSCLVCCLCQNPANFKDLGDLCGPYYPEHCLPKKKPK
E1BXD2(1385-1610)   LG---PVVSKALSAACLVCCLCRSPANYKDLGDLCGPYYPEDCLPKKKSR
G3PEF0(1592-1819)   QG---PLVKRDLIDRCLTCCLCGKPANYRDLGDLCGPYYTEDGVPRKLLT
H3D328(1531-1756)   QG---PSVEKNQTDSCLTCSLCGKPANYRDLGDLCGPYYAEENIPRKLLT
E7F726(1703-1936)   QG---PLVNKSLSDRCLTCCLCGKPANYRELGDLCGPYYPEDCIPRKTLS
C3Z7L5(1697-1932)   IGSAHP--THPVTHAKWVCSFCGKSPNYKELGDLFGPYYLEGKGKRPLLT
E2BIR7(1229-1457)   LS---SRYDAHTPDSTWVCVFCKQGPHSIIPGDPSRPHPNLAGPHIASGT
E2B1Q5(1212-1434)   LS---SRYDAHTTDSTWVCVFCKQGPHSVIPGDPSRPHPNLAGPHIATGT
F4W5Y0(1113-1335)   LS---SRYDAHTTDSTWICVFCKQGPHSVIPGDSSRPHPNLAGPHIATGT
H9HN29(1142-1364)   LS---SRYDAHTTDSTWICVFCKQGPHSVIPGDSSRPHPNLAGPHIATGT
H9K8F1(1148-1369)   LS---SRYDAHTTDSTWVCVLCKQGPHSVIPGDPARPHPNLAGPHIAPGT
G1MZ73(1318-1538)   VG---AQP-GS-----------RSPANYKDLGDLCGPYYPEDCLPKKKSR
F6TA33(1577-1806)   LG---PLVCKAVPSGCLVCCLCRNPANFKDLGDLCGPYYPMDSLPNKKGR
G3X2Q6(1102-1337)   LG---PVVSKTLSAACLVCCLCQNPANYKDLGDLCGPYYPEDCLPKKKSR
G5BMW5(1673-1926)   LG---PVVSKALSTSCLVCCLCQNPANFKDLGDLCGPYYPEHCLPKKKPK
F6R4X5(1594-1827)   LG---PVVSKTLSAACLVCCLCQNPANYKDLGDLCGPYYPEDCLPKKKSR
F7B730(1669-1926)   LG---PVVSKALSTSCLVCCLCQNPANFKDLGDLCGPYYPEHCLPKKKPK
E2RE72(1543-1784)   LG---PVVSKALSTSCLVCCLCQNPANFKDLGDLCGPYYPEHCLPKKKPK
H0Y065(1671-1929)   LG---PVVSKALSTSCLVCCLCQNPANFKDLGDLCGPYYPEHCLPKKKPK
H9G548(1666-1853)   LG---PVVSKTLNATCLVCCLCRNPANYKDQGDLCGPYYPEDCLPKKKSR
H3AX41(1594-1783)   LG---PLVLKTLNDGCLVCCLCGRPANYKELGDLCGPYYPASFLLAKKSA
G1NTH3(1623-1838)   LG---PVVSKALSTSCLVCCLCQNPANFKDLGDLCGPYYPEHCLPKKKPK
G3HHP8(1539-1764)   LG---PVVSKALITSCLVCCLCQNPANFKDLGDLCGPYYPEHCLPKKKPK
Q6AWG9(967-1203)    LS---NRYDADTTDSTWICVFCKRGPHKLGLGDLFGPYLVTSDCDEYRAA
Q16VV4(907-1120)    LS---TKYDADTTDTSWMCVFCKTGPHKQRLGDLFGPYIISTKSSEFKQS
B3NLN9(1085-1322)   LS---NRYDADTTDSTWICVFCKRGPHKLGLGDLFGPYLVTSDCDEYRAA
B0W5K6(960-1174)    LS---TKYDADTTDASWMCVFCKTGPHKMRLGDLFGPYIISTKSGEFAES
A7UTC0(931-1162)    LS---TKYDADTTDTSWMCVFCKTGPHKFRLGDLFGPYIISTASGEYEQS
```

```
E3XES6(921-1156)    LS---TKYDADTTDISWMCVFCKMGPHKFRLGDLFGPYIISTASDEYRAS
B4HNB7(1026-1264)   LS---NRYDADTTDSTWICVFCKRGPHKLGLGDLFGPYLVTSDCDEYRAA
B4QBU9(866-1104)    LS---NRYDADTTDSTWICVFCKRGPHKLGLGDLFGPYLVTSDCDEYRAA
B4J9G8(1119-1356)   LS---NRYDADTTDSSWICVFCKCGPHKLGLGDLFGPYLVSIDCEEYRTA
B4MYL6(1108-1349)   LS---NRYDADTTDSSWICVFCKRGPHKLGLGDLFGPYLVSIDCDEYRAA
B3MHU9(1048-1286)   LS---NRYDADTTDSTWICVFCKRGPHKLGLGDLFGPYLVTSDCEEYRAA


Q9UGU0(1690-1939)   KNPPPKRA------------------------------------------
G3QDX4(1690-1939)   KNPPPKRA------------------------------------------
H2QLT9(1692-1941)   KNPPPKRA------------------------------------------
H9FZ14(1690-1939)   KNPPPKRA------------------------------------------
G1S175(1688-1939)   KNPPPKRA------------------------------------------
H0XML0(1702-1951)   KNPPPKRA------------------------------------------
I3NC70(1718-1966)   KNPPPKRA------------------------------------------
F7CRS8(1718-1967)   KNPPPKRA------------------------------------------
E2RT08(1689-1938)   KNPPPKRA------------------------------------------
G1P5A9(1705-1953)   KNPPPKRA------------------------------------------
E1B8T3(1699-1947)   KNPPPKRA------------------------------------------
G3U8A2(1705-1954)   KNPPPKRA------------------------------------------
G1LSB5(1679-1927)   KNPPPKRA------------------------------------------
Q9EPQ8(1718-1962)   KNPPPKRS------------------------------------------
G1SYZ5(1632-1880)   KNPPPKRA------------------------------------------
H0VVM3(1651-1885)   KNLPPKRA------------------------------------------
F6Q9R3(1692-1946)   KNPPPKRA------------------------------------------
G3WIE0(1668-1874)   KNPPPKRA------------------------------------------
H0ZHA4(1664-1913)   KNPPPKRA------------------------------------------
E1BXI6(1671-1920)   KNPPPKRA------------------------------------------
G1NHL2(1672-1921)   KNPPPKRA------------------------------------------
G1KC34(1689-1937)   KNPPPKRA------------------------------------------
F7BZK2(1597-1840)   KNPPPKKS------------------------------------------
I3JNR0(1311-1549)   KNQPQVRQ------------------------------------------
D3ZG21(1722-1965)   KNPPPKRS------------------------------------------
G7PFP9(1725-1980)   KNPPPKRA------------------------------------------
H2P4M2(1692-1935)   KNPPPKRA------------------------------------------
H2ZXL0(1628-1862)   KNPPPKKT------------------------------------------
G3I0E6(721-964)     KNPPPKRS------------------------------------------
G5BPR9(1037-1303)   KNLPPKRA------------------------------------------
Q4SCG6(1995-2332)   KNQPQVRQCQATAGTNKTGPNADIISNTLNSVQDTQTQDAYFTQSDYAIG
E7FE16(2019-2336)   KNQPQVRQTLSHFGPAATG---MTSIPTEAIPQDTRLQEPQNVKSSAESD
F1SRF7(1698-1893)   KNPPPKRA------------------------------------------
H9KVS1(1635-1821)   KNPPPKRA------------------------------------------
Q7Z5J4(1672-1906)   LKEKVRPEGTC---------------------------------------
H2QCD6(1667-1901)   LKEKVRPEGTC---------------------------------------
F7E488(1674-1909)   LKEKVRPEGTC---------------------------------------
H2NSX0(1678-1908)   LKEKVRPEGTC---------------------------------------
G1RTB7(1668-1903)   LKEKVRPEGTC---------------------------------------
E1B9X1(1644-1885)   LKEKVRLEGTC---------------------------------------
D4A4Z4(1638-1887)   LKEKVRLEGTL---------------------------------------
Q61818(1640-1889)   LKEKARLEGTL---------------------------------------
G1LF06(1637-1873)   LKEKVRPEGPC---------------------------------------
G3QJR9(1645-1879)   LKEKVRPEGTC---------------------------------------
G3TJ97(1586-1821)   LKEKVRLEGAC---------------------------------------
H0V1B2(1316-1545)   LKEKVRPEGTC---------------------------------------
I3N2I1(1670-1914)   LKEKVRPEGTC---------------------------------------
F7DZK3(1652-1884)   FKEKVRPEGIC---------------------------------------
E1BXD2(1385-1610)   LKEKARVEGE----------------------------------------
G3PEF0(1592-1819)   VCPTESIREDS---------------------------------------
H3D328(1531-1756)   IKHPEHLRNES---------------------------------------
E7F726(1703-1936)   LTHRSEFRQNS---------------------------------------
C3Z7L5(1697-1932)   QTKTPPK-------------------------------------------
E2BIR7(1229-1457)   YTVPA---------------------------------------------
E2B1Q5(1212-1434)   YTVPA---------------------------------------------
F4W5Y0(1113-1335)   YTVPA---------------------------------------------
H9HN29(1142-1364)   YTVPA---------------------------------------------
H9K8F1(1148-1369)   YTVPA---------------------------------------------
G1MZ73(1318-1538)   LKEKARAEGE----------------------------------------
F6TA33(1577-1806)   PKDKFR----I---------------------------------------
G3X2Q6(1102-1337)   LKEKARAEGPG---------------------------------------
G5BMW5(1673-1926)   LKEKVRPEGTC---------------------------------------
F6R4X5(1594-1827)   PKEKARAEGLG---------------------------------------
F7B730(1669-1926)   LKEKVRPESTC---------------------------------------
E2RE72(1543-1784)   LKEKVRPEGTC---------------------------------------
H0Y065(1671-1929)   LKEKVRPEGTC---------------------------------------
H9G548(1666-1853)   LKEKIKVDGLS---------------------------------------
H3AX41(1594-1783)   IKERKLLDASK---------------------------------------
```

```
G1NTH3(1623-1838)   LKEKVRLEGTC---------------------------------------
G3HHP8(1539-1764)   LKEKVRLECAF---------------------------------------
Q6AWG9(967-1203)    VQTPG---------------------------------------------
Q16VV4(907-1120)    QTDEE---------------------------------------------
B3NLN9(1085-1322)   VQAPG---------------------------------------------
B0W5K6(960-1174)    KTDQD---------------------------------------------
A7UTC0(931-1162)    QVDVD---------------------------------------------
E3XES6(921-1156)    QVDVD---------------------------------------------
B4HNB7(1026-1264)   VQTPG---------------------------------------------
B4QBU9(866-1104)    VQAPG---------------------------------------------
B4J9G8(1119-1356)   VQVPAG--------------------------------------------
B4MYL6(1108-1349)   LHSPG---------------------------------------------
B3MHU9(1048-1286)   LQAPG---------------------------------------------


Q9UGU0(1690-1939)   ------------------TEMQSKVKVRHKSASNGS--------------
G3QDX4(1690-1939)   ------------------TEMQSKVKVRHKSASNGS--------------
H2QLT9(1692-1941)   ------------------TEMQSKVKVRHKSASNGS--------------
H9FZ14(1690-1939)   ------------------TEMQSKVKVRHKSASNGS--------------
G1S175(1688-1939)   ------------------TEMQSKVKVRHKSASNGS--------------
H0XML0(1702-1951)   ------------------SEMQSKVKVRHKSASNGS--------------
I3NC70(1718-1966)   ------------------TEMQSKVKVRHKSASNGS--------------
F7CRS8(1718-1967)   ------------------MEMQSKVKVRHKSASNGS--------------
E2RT08(1689-1938)   ------------------TEMQSKVKVRHKSASNGS--------------
G1P5A9(1705-1953)   ------------------TEMQSKVKVRHKSASNGS--------------
E1B8T3(1699-1947)   ------------------AETQSKVKVRHKSASNGS--------------
G3U8A2(1705-1954)   ------------------TEMQSKVKVRHKSASNGS--------------
G1LSB5(1679-1927)   ------------------TEMQSKVKVRHKSASNGS--------------
Q9EPQ8(1718-1962)   ------------------SEMQSKVKVRHKSASNGS--------------
G1SYZ5(1632-1880)   ------------------AEMQSKVKVRHKSASNGS--------------
H0VVM3(1651-1885)   ------------------TEMQSKVKVRHKSASNGS--------------
F6Q9R3(1692-1946)   ------------------TEMQNKVKVRHKSAPNGCA-------------
G3WIE0(1668-1874)   ------------------TEMQNKVKVRHKSASNGCP-------------
H0ZHA4(1664-1913)   ------------------TEMQSKVKVRHKSASNGS--------------
E1BXI6(1671-1920)   ------------------TEMQSKVKVRHKSASNGS--------------
G1NHL2(1672-1921)   ------------------TEMQSKVKVRHKSASNGS--------------
G1KC34(1689-1937)   ------------------TEMQHTVKVRHKSASNGS--------------
F7BZK2(1597-1840)   ------------------SETPRKVKVRHKDTSDGSK-------------
I3JNR0(1311-1549)   ----------------SGREEMMTHVADKHSNAASSSSSSSSS-------
D3ZG21(1722-1965)   ------------------TEMQNKVKVRHKSASNGS--------------
G7PFP9(1725-1980)   ------------------TEMQSKVKVRHKSASNGS--------------
H2P4M2(1692-1935)   ------------------TEMQSKVKVRHKSASNGS--------------
H2ZXL0(1628-1862)   ------------------TETQGRVKVRHKSLSDGS--------------
G3I0E6(721-964)     ------------------TEMQSKVKVRHKSTSNGS--------------
G5BPR9(1037-1303)   ------------------TEMQSKVKVRHKSASNGS--------------
Q4SCG6(1995-2332)   MDSTSLAAALRPVSTATREETMTHLAGRFSNTASSSSPSSSSSSSHALSK
E7FE16(2019-2336)   CTVSQATNTTSPATIIGTVSPMP-LEMPFQNAIASSSTARVTTHAWDTAA
F1SRF7(1698-1893)   ------------------TEMQSKVKVRHKSASNGS--------------
H9KVS1(1635-1821)   ------------------TEMQSKVKVRHKSASNGS--------------
Q7Z5J4(1672-1906)   ------------------EEASLPLERTLKGPECAAAA------------
H2QCD6(1667-1901)   ------------------EEALLPLERTLKGPECAAAA------------
F7E488(1674-1909)   ------------------EEASLPLERTLKGPECAATT------------
H2NSX0(1678-1908)   ------------------EAS-LPLE-TLK-PECAAAA------------
G1RTB7(1668-1903)   ------------------EEASLPLERTLKGPDCAAAA------------
E1B9X1(1644-1885)   ------------------EEASLPLERTLKGFECPAAASS----------
D4A4Z4(1638-1887)   ------------------EEASLPLERTLKGLECAASTTAATP-------
Q61818(1640-1889)   ------------------EEASLPLERTLKGLECSASTTAAAP-------
G1LF06(1637-1873)   ------------------EEASPPLERTLKDLECAATAA-----------
G3QJR9(1645-1879)   ------------------EEASLPLERTLKGPECAAAA------------
G3TJ97(1586-1821)   ------------------EEASLPLERTLRGLECAVAAT-----------
H0V1B2(1316-1545)   ------------------EEALLPLERTLKGLEC----------------
I3N2I1(1670-1914)   ------------------EEASPPLERTLK-VECAAPAPVAAA-------
F7DZK3(1652-1884)   ------------------EEASLPLERTLKGLECAAAAT-----------
E1BXD2(1385-1610)   ------------------EGGAAERPRGAAESSWVAG-------------
G3PEF0(1592-1819)   ------------------QKTNDPNGSSSEEPSSSKNEGES---------
H3D328(1531-1756)   ------------------QKTDDGHSGNDEVPGCSSSEGAE---------
E7F726(1703-1936)   ------------------NCANETESSSIVKNTQSVCEEAI---------
C3Z7L5(1697-1932)   -----------------QSADLKENKVRKSRSRNPS--------------
E2BIR7(1229-1457)   ------------------GVSGDLFGPYLIGKDRLE--------------
E2B1Q5(1212-1434)   ------------------GVLGDLFGPYLIGK------------------
F4W5Y0(1113-1335)   ------------------GVLSDLFGPYLIGK------------------
H9HN29(1142-1364)   ------------------GVLSDLFGPYLIGK------------------
H9K8F1(1148-1369)   ------------------GVLSDLFGPYLIGK------------------
G1MZ73(1318-1538)   ------------------EGGAAERPRGVAESSWAAG-------------
F6TA33(1577-1806)   --------------------AEAPAEKAAKVSDSVCTVGS----------
G3X2Q6(1102-1337)   ------------------EE-AAPLERTLRGLESPCPAGP----------
```

```
G5BMW5(1673-1926)  -------------------EEAPLPLERTLKGLECVA----------------
F6R4X5(1594-1827)  -------------------EDAAAPPDRTLRALESPCPAG-------------
F7B730(1669-1926)  -------------------EETSLPLERTLKGPECAAAA--------------
E2RE72(1543-1784)  -------------------EEASPPLERTLKDLECVAATG-------------
H0Y065(1671-1929)  -------------------EEASLPLERTLKGLECAAAA--------------
H9G548(1666-1853)  ----------------------EEPPSLSLLKVSTDNNCSG------------
H3AX41(1594-1783)  -------------------AERLKPLPSESVATETNDKSLLS-----------
G1NTH3(1623-1838)  -------------------EEASLPLERTLKGLECAAAA--------------
G3HHP8(1539-1764)  -------------------EETSLPLERTLKGLECAAS---------------
Q6AWG9(967-1203)   -------------AQDIDGMFVNKRRREDMVKGQERNL---------------
Q16VV4(907-1120)   -------------------YFNVKRTRESLQSKLIK-----------------
B3NLN9(1085-1322)  -------------AQDIDGLFVNKRRREDMVKGQERNL---------------
B0W5K6(960-1174)   -------------------YFSEKRTRESLASKLVQP----------------
A7UTC0(931-1162)   -------------------FFSVRRTREDLESSQAKQR---------------
E3XES6(921-1156)   -------------------YFSVKRSRDSMESTQAKER---------------
B4HNB7(1026-1264)  -------------AQDIDGLFVNKRRREDMVKGQERNL---------------
B4QBU9(866-1104)   -------------AQDIDGLFVNKRRREDMVKGQERNL---------------
B4J9G8(1119-1356)  -------------SNDIDGLFVSKRKRADMVKLQERNL---------------
B4MYL6(1108-1349)  -------------VQDIDGLFVSKRKRGDMVKAQQRNL---------------
B3MHU9(1048-1286)  -------------VQDIDGLFVSKRRREDMVKVQERNL---------------


Q9UGU0(1690-1939)  -----------------------------------KTDTEEEEEEQQQQQKEQ
G3QDX4(1690-1939)  -----------------------------------KTDTEEEEEEQQQQQKEQ
H2QLT9(1692-1941)  -----------------------------------KTDTEEEEEEQQQQQKEQ
H9FZ14(1690-1939)  -----------------------------------KTDTEEEEEEQQQQQKEQ
G1S175(1688-1939)  -----------------------------------KTDTEEEEEEQQQQQKEQ
H0XML0(1702-1951)  -----------------------------------KTDTEEEEEEQQQQQKEQ
I3NC70(1718-1966)  -----------------------------------KTDTEEEEEEQQQQ-KEQ
F7CRS8(1718-1967)  -----------------------------------KTDTEEEEEEQQQQQKEQ
E2RT08(1689-1938)  -----------------------------------KTDTEEEEEEQQQQQKEQ
G1P5A9(1705-1953)  -----------------------------------KTDTEEEEEE-QQQQKEQ
E1B8T3(1699-1947)  -----------------------------------KTDTEEEEEEQQQQ-KEQ
G3U8A2(1705-1954)  -----------------------------------KTDTEEEEEERQQQQKEQ
G1LSB5(1679-1927)  -----------------------------------KTDT-EEEDEQQQQKEQ
Q9EPQ8(1718-1962)  -----------------------------------KTDTEEEEEEQQQQ-KEQ
G1SYZ5(1632-1880)  -----------------------------------KTDTEEEEEEQQQQ-KEQ
H0VVM3(1651-1885)  -----------------------------------KTDT--------------
F6Q9R3(1692-1946)  -----------------------------------KTDTEEEEEEEEEEQKEQ
G3WIE0(1668-1874)  -----------------------------------KTDTEEEEEEEEEE----
H0ZHA4(1664-1913)  -----------------------------------KTDTEEEEEEQQQQ-KEQ
E1BXI6(1671-1920)  -----------------------------------KTDTEEEEEEQQQQ-KEQ
G1NHL2(1672-1921)  -----------------------------------KTDTEEEEEEQQQQ-KEQ
G1KC34(1689-1937)  -----------------------------------KTDTEEEEEEQQQ--KEQ
F7BZK2(1597-1840)  -----------------------------------SDSDEEEEEEEPQQAREQ
I3JNR0(1311-1549)  --------------------------------------------YEAQQRPQH
D3ZG21(1722-1965)  -----------------------------------KTDTEEEEEEQQQQQKEQ
G7PFP9(1725-1980)  -----------------------------------KTDTEEEEEEQQQQQKEQ
H2P4M2(1692-1935)  -----------------------------------KTDTEEEEEEQQQQQKEQ
H2ZXL0(1628-1862)  -----------------------------------KTDSEDEP-------KEQ
G3I0E6(721-964)    -----------------------------------KTDTEEEEEEQQQQQKEQ
G5BPR9(1037-1303)  -----------------------------------KTDTEEEEEEQQ---KEQ
Q4SCG6(1995-2332)  PTSLTWDTSLDIHPVPELKKEADADGEPQWPRKQPQLQPPPDEAQQRPQH
E7FE16(2019-2336)  ELESG-----------------LGTSNLQETDAEIASKQLQVESSQQRPQH
F1SRF7(1698-1893)  -----------------------------------KTDTEEEEEEQQQQQKEQ
H9KVS1(1635-1821)  -----------------------------------KTDTEEEE----------
Q7Z5J4(1672-1906)  --------------------------------------------TAGKPPRP-
H2QCD6(1667-1901)  --------------------------------------------TAGKPPRP-
F7E488(1674-1909)  --------------------------------------------AAGKPPRP-
H2NSX0(1678-1908)  --------------------------------------------AAGKPPRP-
G1RTB7(1668-1903)  --------------------------------------------AAGKPPRP-
E1B9X1(1644-1885)  ----------------------------------------AATTTTGKPPRP-
D4A4Z4(1638-1887)  -----------------------------------TTTTITTTTTLGRLSRP-
Q61818(1640-1889)  -----------------------------------TTATITTPTALGRLSRP-
G1LF06(1637-1873)  ------------------------------------------P---GKPPRP-
G3QJR9(1645-1879)  --------------------------------------------TAGKPPRP-
G3TJ97(1586-1821)  ---------------------------------------------AGKPPRP-
H0V1B2(1316-1545)  ----------------------------------------------GKPPRL-
I3N2I1(1670-1914)  ------------------------------------AAAATTTTTAGKPPRP-
F7DZK3(1652-1884)  ------------------------------------------AT--GKPPRP-
E1BXD2(1385-1610)  ----------------------------------------------GRAGRP-
G3PEF0(1592-1819)  --------------------------------------------STEKEDN--
H3D328(1531-1756)  -------------------------------------------GGAEKDG---
E7F726(1703-1936)  --------------------------------------------HQEKANEGH
C3Z7L5(1697-1932)  ---------------------------------------EVSGSEPKPGPSGL
E2BIR7(1229-1457)  -------------------------------------------DGKERLEDGI
```

```
E2B1Q5(1212-1434)   -------------------------------------------ERLEDGI
F4W5Y0(1113-1335)   -------------------------------------------ERLEDGI
H9HN29(1142-1364)   -------------------------------------------ERLEDGI
H9K8F1(1148-1369)   -------------------------------------------ERLEDGI
G1MZ73(1318-1538)   -------------------------------------------GRAGRQ-
F6TA33(1577-1806)   -------------------------------------------GRAPCSD
G3X2Q6(1102-1337)   -------------------------------------------GKPPRP-
G5BMW5(1673-1926)   ---------------------------------ATPAAAATGSGKPPRL-
F6R4X5(1594-1827)   --------------------------------------------KPPRPD
F7B730(1669-1926)   -----------------------------------------TAGKPPRP-
E2RE72(1543-1784)   ---------------------------------------ATAAGKPPRP-
H0Y065(1671-1929)   -----------------------------------------APGKPSRP-
H9G548(1666-1853)   -------------------------------------------GKSPRL-
H3AX41(1594-1783)   -----------------------------------------IEENQPRL-
G1NTH3(1623-1838)   ------------------------------------------ASKAPRP-
G3HHP8(1539-1764)   --------------------------------TITTITTTTTLGRLSRP-
Q6AWG9(967-1203)    ----------------------------------------PAVPATLANI
Q16VV4(907-1120)    -----------------------------------------------KEE
B3NLN9(1085-1322)   ----------------------------------------PAVPATLATI
B0W5K6(960-1174)    -----------------------------------------------KVE
A7UTC0(931-1162)    ----------------------------------------RAAEKLKEQQ
E3XES6(921-1156)    ----------------------------------------RMAEQQKQQR
B4HNB7(1026-1264)   ----------------------------------------PAVPATLAYI
B4QBU9(866-1104)    ----------------------------------------PAVPATLANI
B4J9G8(1119-1356)   ----------------------------------------PVVPATLANI
B4MYL6(1108-1349)   ----------------------------------------PVVQASLANI
B3MHU9(1048-1286)   ----------------------------------------PVVPATLATI


Q9UGU0(1690-1939)   RSLAAHPRFKRRHRSEDCGGGPRSLSRGLPCKKAATEGSS----EKTVLD
G3QDX4(1690-1939)   RSLAAHPRFKRRHRSEDCGGGPRSLSRGLPCKKAATEGSS----EKTVLD
H2QLT9(1692-1941)   RSLAAHPRFKRRHRSEDCGGGPRSLSRGLPCKKAATEGSS----EKTVLD
H9FZ14(1690-1939)   RSLAAHPRFKRRHRSEDCGGGPRSLSRGLPCKKAATEGSS----EKTVLD
G1S175(1688-1939)   RSLATHPRFKRRHRSEDCGGGPRSLSRGLPCKKAATEGSS----EKTVLD
H0XML0(1702-1951)   RSLATHPRFKRRHRSEDCGGGPRSLSRGLPCKKAATEGSS----EKTALD
I3NC70(1718-1966)   RSLAAHPRFKRRHRSEDCGGGPRSLSRGLPCKKAATEGSS----EKTVLD
F7CRS8(1718-1967)   RSLAAHPRFKRRHRSEDCGGGPRSLSRGLPCKKATTEGSS----EKTVLD
E2RT08(1689-1938)   RSLAAHPRFKRRHRSEDCSGGPRSLSRGLPCKKATAEGSS----DKTALD
G1P5A9(1705-1953)   RSLAAHPRFKRRHRSEDCGGGPRSLARGLPCKKATTEGGS----EKTVLD
E1B8T3(1699-1947)   RSLAAHPRFKRRHRSEDCAGGPRSLSRGLPCKKATTEGSS----EKTVLD
G3U8A2(1705-1954)   RSLAAHPRFKRRHRSEDCGGGPRSLSRGLPCKKATTEGSS----EKTALD
G1LSB5(1679-1927)   RSLAAHPRFKRRHRSEDCGGGPRSLSRGLPCKKATAEGSS----EKTALD
Q9EPQ8(1718-1962)   RSLAAHPRFKRRHRSEDCGGGPRSLSRGLPCKKAATEGSS----EKTVSD
G1SYZ5(1632-1880)   RSLAAHPRFKRRHRSEDCGGGPRALSRGLPCKKAAAEGSS----EKTALD
H0VVM3(1651-1885)   --LAAHPRFKRRHRSEDCGGGPRSLSRGLPCKKAATEGSS----EKTVLD
F6Q9R3(1692-1946)   RSLAAHPRFKRRHRSEDCSGTPRSLSRGIPCKKATIEGGSGSGNEKTPSD
G3WIE0(1668-1874)   --------------------------------------------EPQPSD
H0ZHA4(1664-1913)   RSLAAHPRFKRRHRSEDCSGASRSLSRGASCKKATTDGGSGG--EKTPLD
E1BXI6(1671-1920)   RSLAAHPRFKRRHRSEDCSGASRSLSRGASCKKATTEGGSGG--EKTPLD
G1NHL2(1672-1921)   RSLAAHPRFKRRHRSEDCSGASRSLSRGASCKKATTEGGSGG--EKTPLD
G1KC34(1689-1937)   RSLTAHPRFKRRHRSEDCACASRSLSRGAACKKATTEVGNVG--EKTPSD
F7BZK2(1597-1840)   RSLTAHPRYKRRNRSGDCTSSRLT----VPSHRKTTDPSE-----LTPMD
I3JNR0(1311-1549)   RKLTSHPRFKRRHKSSE------DSPRMVPANSKASLPFQ----------
D3ZG21(1722-1965)   RSLAAHPRFKRRHRSEDCGGGPRSLSRGLPCKKAAAEGSS----EKTASD
G7PFP9(1725-1980)   RSLAAHPRFKRRHRSEDCGGGPRSLSRGLPCKKAATEGSS----EKTVLD
H2P4M2(1692-1935)   RSLAAHPRFKRRHRSEDCGGGPRSLSRGLPCKKAATEGSS----EKTVLD
H2ZXL0(1628-1862)   RSLPAHPRFKRRHRSEDLG-SPRSIARGALCRKPPSDTCG-----KSPTD
G3I0E6(721-964)     RSLAAHPRFKRRHRSEDCGGGPRSLSRGLPCKKAATEGSC----EKAVLD
G5BPR9(1037-1303)   RSLAAHPRFKRRHRSEDCGGGPRSLSRGLPCKKAATEGSS----EKTILD
Q4SCG6(1995-2332)   RKLTSHPRFKRRHKSSE------DCPRMVPSNSKASLPFQ----------
E7FE16(2019-2336)   RKLTSHPRFKRRHKSSE------DLPRTVPINSKASLPFQ----------
F1SRF7(1698-1893)   RSLAAHPRFKRRHRSEDCGGGPRSLSRGLPCKKATTEGSS----EKTALD
H9KVS1(1635-1821)   RSLAAHPRFKRRHRSEDCGGGPRSLSRGLPCKKAATEGSS----EKTVLD
Q7Z5J4(1672-1906)   --DGPADPAKQGPLRTSARGLSRRLQSCYCCDGREDGGE---------EA
H2QCD6(1667-1901)   --DGPADPAKQGPLRTSARGLSRRLQSCYCCDGREDGGE---------EA
F7E488(1674-1909)   --DGPADPAKQGPLRTSARGLSRRLQSCYCCDGREDGGE---------EA
H2NSX0(1678-1908)   --DGPADPAKQGPLRTSARGLSRRLQSCYCCDGREDGGE---------EA
G1RTB7(1668-1903)   --DGSADPAKQGPLRTSARGLSRRLQSCYCCDGREDGGE---------EA
E1B9X1(1644-1885)   --DGPADPAKQGSLRTSARGLSRRLQSCYCCDGRGDGSE---------EA
D4A4Z4(1638-1887)   --DGPADPAKQGSLRTSARGLSRRLQSCYCCDGQGDGGE---------EV
Q61818(1640-1889)   --DGPADPAKQGPLRTSARGLSRRLQSCYCCDGQGDGGE---------EV
G1LF06(1637-1873)   --EGPADPAKQGSLRTSARGLSRRLQSCYCCDGRGDGGE---------EA
G3QJR9(1645-1879)   --DGPADPAKQGPLRTSARGLSRRLQSCYCCDGREDGGE---------EA
G3TJ97(1586-1821)   --DGPADPAKQGSLRTSARGLSRRLQSCYCCDGQGDGGE---------EA
H0V1B2(1316-1545)   --DGPADPAKQGSLRTSARGLSRRLQSCYCCDGRGDGGE---------EM
I3N2I1(1670-1914)   --DGPADPAKQGSLRTSARGLSRRLQSCYCCDGRGDGGE---------EV
```

```
F7DZK3(1652-1884)  --DGPADPAKQGSVRTSARGLSRRLQSCYCCDGRGDSGE---------EV
E1BXD2(1385-1610)  --EGAAEPGKPSVLRSSPRGVFRRLQSCYCCDERTEGE----------EA
G3PEF0(1592-1819)  --SETTTQEEGGPRRITLRERFRRMKQLRA--ISAAASS-----------
H3D328(1531-1756)  --PETSTQE--GPRRLTLQERLRRMRQLQA--GGTRSPS-----------
E7F726(1703-1936)  SRRSKRAIRELSRTRPSSRMRFKRLLILQSRLSGASPPA-----------
C3Z7L5(1697-1932)  SSPANTPLSKRRHRSQ---------SGGEKGRKAAHKGGA----------
E2BIR7(1229-1457)  LSADEQEITREQKKGG---KNKRSLRYAGLADQFSAKMGKK--------K
E2B1Q5(1212-1434)  LSADEQEITTEQKKGG---KNKRSLRYAGLADQFSAKMGKK--------K
F4W5Y0(1113-1335)  LSADEQEITTEQKKGG---KNKRSLRYAGLADQFSAKMGKK--------K
H9HN29(1142-1364)  LSADEQEITTEQKKGG---KNKRSLRYAGLADQFSAKMGKK--------K
H9K8F1(1148-1369)  LSADEQEITTEQKKGG---KNKRSLRYAGLADQFTAKMGKK--------K
G1MZ73(1318-1538)  --EAAAEPGKPGVLRSSPRGVFRRLQSCYCCDERTEGE----------EA
F6TA33(1577-1806)  --VGELDPSKHNNLRTSARGMLRKLPSCYCCSKTVEIAE-----------
G3X2Q6(1102-1337)  --EAPAEAAKPSSLRSSSRGLYRKLQSCYCCDEQRTEDE---------EA
G5BMW5(1673-1926)  --DAPADPAKQGSLRTSARGLSRRLQSCYCCDGRGDGGD---------EM
F6R4X5(1594-1827)  --VAAADSTKPSALRSSSRGLYRKLQSCYCCDEQRTTED---------EE
F7B730(1669-1926)  --DGPADLAKQGPLRTSARGLSRRLQSCYCCDGREDGGE---------EA
E2RE72(1543-1784)  --EGPADPAKQGSLRTSARGLSRRLQSCYCCDARGDGGE---------EA
H0Y065(1671-1929)  --DGPADPAKQGSLRTSARGLSRRLQSCYCCDGRGDGGE---------EV
H9G548(1666-1853)  --DTGAESAKQSTLRSSSRGMFRKLQSCYCCDERTEGEDT--------AA
H3AX41(1594-1783)  --DSTSDITKTSGVRSSSRGLCRKQQSCYCCDSKSEDTE-----------
G1NTH3(1623-1838)  --DGSADPAKQGSLRTSARGLSRRLQSCYCCDGRGDGGE---------EV
G3HHP8(1539-1764)  --DGPADPAKQGSLRTSARGLSRRLQSCYCCDGQGDGGE---------EV
Q6AWG9(967-1203)   MQAPKISMHKRKRKQ----THDSSISYSDDPNESRSQCSS----------
Q16VV4(907-1120)   PVAGTSNSKSKKRKPG---TEAAANPPAVPSTSVAPPDPA----------
B3NLN9(1085-1322)  MQAPKISMHKRKRKQ----THDSSISYSDDPNETRSQCSS----------
B0W5K6(960-1174)   PTAG-SGTKSKKRKSV---EPTASTSSAVAAAPPPLTATV----------
A7UTC0(931-1162)   QQGGP---KGKKRKNAAG--AAAAVPGAPAGPSSAKAMALK---------
E3XES6(921-1156)   QQAASGSGKGRKRKN-----AASATNNAQAPSTSNCPAVLK---------
B4HNB7(1026-1264)  MQAPKISMHKRKRKQ----THDSSISYSDDPNESRSQCSS----------
B4QBU9(866-1104)   MQAPKISMHKRKRKQ----THDSSISYSDDPNESRSQCSS----------
B4J9G8(1119-1356)  MQAPKITMHKRKRKQ----THDSVYSCSDDPNE--SQCSS----------
B4MYL6(1108-1349)  MQAPKISMHKRKRKQP---AQDCNNSFSDDQFDMQSRCSSS--------M
B3MHU9(1048-1286)  MQAPKISMHKRKRKQ----THDSQVSYSDDPNESQSQCSS----------
```

```
                                                   3  4
Q9UGU0(1690-1939)  SKPSVPTTSEGGPELEL-QIPELPLDSNEFWVHEGCILWANGIYLVCGRL
G3QDX4(1690-1939)  SKPSVPTTSEGGPELEL-QIPELPLDSNEFWVHEGCILWANGIYLVCGRL
H2QLT9(1692-1941)  SKPSVPTTSEGGPELEL-QIPELPLDSNEFWVHEGCILWANGIYLVCGRL
H9FZ14(1690-1939)  SKPSVPTTSEGGPELEL-QIPELPLDSNEFWVHEGCILWANGIYLVCGRL
G1S175(1688-1939)  SKPSVPTTSEGGPELEL-QIPELPLDSNEFWVHEGCILWANGIYLVCGRL
H0XML0(1702-1951)  SKPSVPTTSEGGPELEL-QIPELPLDSNEFWVHEGCILWANGIYLVCGRL
I3NC70(1718-1966)  TKPSVPTTSEGGPELEL-QIPELPLDSNEFWVHEGCILWANGIYLVCGRL
F7CRS8(1718-1967)  SKPSVPTTSEGGPELEL-QIPELPLDSNEFWVHEGCILWANGIYLVCGRL
E2RT08(1689-1938)  SKPSAPTTSEGGPELEL-QIPELPLDSNEFWVHEGCILWANGIYLVCGRL
G1P5A9(1705-1953)  SKPSVPTTSEGGPELEL-QIPELPLDSNEFWVHEGCILWANGIYLVCGRL
E1B8T3(1699-1947)  SKPSVPTTSEGGPELEL-QIPELPLDSNEFWVHEGCILWANGIYLVCGRL
G3U8A2(1705-1954)  SKPCVPTTSEGGPELEL-QIPELPLDSNEFWVHEGCILWANGIYLVCGRL
G1LSB5(1679-1927)  PKPSVPTTSEGGPELEL-QIPELPLDSNEFWVHEGCILWANGIYLVCGRL
Q9EPQ8(1718-1962)  TKPSVPTTSEGGPELEL-QIPELPLDSNEFWVHEGCILWANGIYLVCGRL
G1SYZ5(1632-1880)  SKPPVPATSEGGPEPEL-QIPELPLDSNEFWVHEGCILWANGIYLVCGRL
H0VVM3(1651-1885)  TKPSVPTTSEGGPELEL-QIPELPLDSNEFWVHEGCILWANGIYLVCGRL
F6Q9R3(1692-1946)  SKPAGPVSEGAGPELEL-HIPELPLDSNELWVHEGCVLWANGIYLVCGRL
G3WIE0(1668-1874)  SKPAGPTLEGAGPELEL-HIPELPLDSNELWVHEGCVLWANGIYLVCGRL
H0ZHA4(1664-1913)  SKPSVPTS-EGGTELEL-QIPELPLDSNEFWVHEGCILWANGIYLVCGRL
E1BXI6(1671-1920)  SKPSMPTS-EGGTELEL-QIPELPLDSNEFWVHEGCILWANGIYLVCGRL
G1NHL2(1672-1921)  SKPSMPTS-EGGTELEL-QIPELPLDSNEFWVHEGCILWANGIYLVCGRL
G1KC34(1689-1937)  SKPSMPTS-EGGPELEL-QIPELPLDSNEFWVHEGCILWANGIYLVCGRL
F7BZK2(1597-1840)  S--SGPSTAEAGPEQGV-QIPQLPLDSNEFWMHEGCVLWANGVYFVCGRL
I3JNR0(1311-1549)  --PPPPALDSLGPLAQLAQLPQMPMDPEELWVHEGCIVWTSGVYLVNGRL
D3ZG21(1722-1965)  TKPSVPTTSEGGPELEL-QIPELPLDSNEFWVHEGCILWANGIYLVCGRL
G7PFP9(1725-1980)  SKPSVPTTSEGGPELEL-QIPELPLDSNEFWVHEGCILWANGIYLVCGRL
H2P4M2(1692-1935)  SKPSVPTTSEGGPELEL-QIPELPLDSNEFWVHEGCILWANGIYLVCGRL
H2ZXL0(1628-1862)  SNPQAPPT-EVSSEVGL-QIPELPLDSNEFWVHEACILWANGVYLVCGKL
G3I0E6(721-964)    TKPSVPTTSEGGPELEL-QIPELPLDSNEFWVHEGCILWANGIYLVCGRL
G5BPR9(1037-1303)  TKPSMPTTSEGGPEPEL-QIPELPLDSNEFWVHEGCILWANGIYLVCGRL
Q4SCG6(1995-2332)  --PPPPALDSLGPLAQLAQLPKMPMDPEELWVHEGCIVWTSGVYLVSGRL
E7FE16(2019-2336)  --PPPPSLDALGPMAQLAQLPLVPLDPEELWVHESCMVWTSGIYLVNGRL
F1SRF7(1698-1893)  SKPSVSTTSEGGPELEL-QIPELPLDSNEFWVHEGCILWANGIYLVCGRL
H9KVS1(1635-1821)  SKPSVPTTSEGGPELEL-QIPELPLDSNEFWVHEGCILWANGIYLVCGRL
Q7Z5J4(1672-1906)  AP-ADKGRKHECSK-EAPAEPGG--EAQEHWVHEACAVWTGGVYLVAGKL
H2QCD6(1667-1901)  AP-ADKGRKHECSK-EAPAEPGG--EAQEHWVHEACAVWTSGVYLVAGKL
F7E488(1674-1909)  AP-ADKGRKHECSK-EAPAEPGG--EAQEHWVHEACAVWTGGVYLVAGKL
H2NSX0(1678-1908)  AP-ADKGRKHECSK-EAPAEPGG--EAQEHWVHEACAVWTSGVYLVAGKL
G1RTB7(1668-1903)  AP-ADKGRKHECSK-EAPAEPGG--EAQEHWVHEACAVWTSGVYLVAGKL
E1B9X1(1644-1885)  AP-ADKSRKHECSK-EPPAEPGG--DTQEHWVHEACAVWTGGVYLVAGKL
```

```
D4A4Z4 (1638-1887)   AP-ADKSRKHECSK-EAPAEPGG--DTQEHWVHEACAVWTSGVYLVAGKL
Q61818 (1640-1889)   AQ-ADKSRKHECSK-EAPTEPGG--DTQEHWVHEACAVWTSGVYLVAGKL
G1LF06 (1637-1873)   AP-ADKSRKHECSK-EPPAEPGG--DTQEHWVHEACAVWTGGVYLVAGKL
G3QJR9 (1645-1879)   AP-ADKGRKHECSK-EAPAEPGG--EAQEHWVHEACAVWTGGVYLVAGKL
G3TJ97 (1586-1821)   AP-ADKSRKHECSK-EDSAEPSG--GTQEHWVHEACAVWTGGVYLVAGKL
H0V1B2 (1316-1545)   AP-VDKSRKHECVK-EAPAEPVG--DTQEHWVHEACAVWTGGVYLVAGKL
I3N2I1 (1670-1914)   AA-ADKSRKHECSR-EGPAEPGG--DTQEHWVHEACAVWTSGVYLVAGKL
F7DZK3 (1652-1884)   AP-ADKSRKHECSK-EPPAEPGG--DTQEHWVHEACAVWTGGVYLVAGKL
E1BXD2 (1385-1610)   AE---KPRRHECTKAESPPQPEPAGDTQEHWLHEACAVWTAGVFLVAGKL
G3PEF0 (1592-1819)   ---DREGGDSMFERLQMEAEA------KEHWAHENCAIWTKGVIMVAGRL
H3D328 (1531-1756)   ---DPEGKDSMLQRLRVQAEA------NEHWTHENCAIWTKGIIMVAGRL
E7F726 (1703-1936)   ---GVEGIETSLQRLQMEAEE------KEHWAHEACAVWTTGIVLVADKL
C3Z7L5 (1697-1932)   KVAARKAGKEGGSPPCSLEAVVVPMYLDEVWTHDQCAVWASGVFIMGGRL
E2BIR7 (1229-1457)   RNSVESNTN--AMFTGMTMLPG-EEQRWEVWLHEQCAVWAAGVYMAGGRV
E2B1Q5 (1212-1434)   RNSIESNTN--TIFTGMTLYPGGEEQRWEVWLHEQCAVWAAGVYMAGGRV
F4W5Y0 (1113-1335)   RNSVESNTN--VIFTGMTLHPGGEEQRWEVWLHEQCAVWAAGVYMAGGRV
H9HN29 (1142-1364)   RNSVESNTN--VIFTGMTLHPGGEEQRWEVWLHEQCAVWAAGVYMAGGRV
H9K8F1 (1148-1369)   RNSIESNTN--AMFTGMTVLPG-EEQRWEVWLHEQCAVWAAGVYMAGGRV
G1MZ73 (1318-1538)   AE---KPRRHECTKAESPPQPEPAGDTQEHWLHEACAVWTAGVFLVAGK-
F6TA33 (1577-1806)   ---TEKPRRHQCNKALEPPTP--ELETQEHWVHEACAVWTSGVYLVAGKL
G3X2Q6 (1102-1337)   PA-ADKPRKHECSK-VEAPAPELSGDTQEHWVHEGCAIWTAGVYLVAGKL
G5BMW5 (1673-1926)   AP-VDKSRKHECSK-EAPSEP--VGDTQEHWVHEACAVWTGGVYLVAGKL
F6R4X5 (1594-1827)   AA-ADK----------------------EHWVHEGCAIWTAGVYLVAGKL
F7B730 (1669-1926)   AP-ADKGRRHECSK-EAPAEPGG--EAQEHWVHEACAVWTGGVYLVAGKL
E2RE72 (1543-1784)   AP-ADK----------------------EHWVHEACAVWTGGVYLVAGKL
H0Y065 (1671-1929)   AP-ADKSRKHECSK-EAPAEPGG--DTQEHWVHEACAVWTSGVYLVAGKL
H9G548 (1666-1853)   ATASEKPRRHECSKADVP-LPDPAGDTQEHWVHEACAIWTSGVYLVAGKL
H3AX41 (1594-1783)   ---PEKSKSSQCNQVDSQLGKP-VADSKEHWFHESCVIWTQGVYLVAGKL
G1NTH3 (1623-1838)   APVADKTRKHECSK-EAPAEPGG--DTQEHWVHEACAVWTGGVYLVAGKL
G3HHP8 (1539-1764)   AP-ADKSRKHECSK-EAPAEPGG--DTQEHWVHEACAVWTSGVYLVAGKL
Q6AWG9 (967-1203)    VDLLDCSTES-KFVETFRGMGKTSENGFEVWLHEDCAVWSNDIHLIGAHV
Q16VV4 (907-1120)    QFP----------DDIFYGMIKAGDDSYEVWMHEDCLVWAPGVHIIGTRV
B3NLN9 (1085-1322)   VDPLDCSTES-KFVETFRGMGKTSENGFEVWLHEDCAVWSNDIHLIGAHV
B0W5K6 (960-1174)    PPPP---------PDMFYGMVKAGEDSYEVWLHEDCLVWAPGVHIIGTRI
A7UTC0 (931-1162)    QEPCDDVVDR-SACEIFYGMVKASDNTYEVWTHEDCLVWAPGVYMVGTRI
E3XES6 (921-1156)    EEKKDDDQGQSSHTDVFYGMMKASDSTYEVWTHEDCLVWAPGVYMVGTRV
B4HNB7 (1026-1264)   VDPLDCTTES-KFVETFRGMGKTSENGFEVWLHEDCAVWSNDIHLIGAHV
B4QBU9 (866-1104)    VDPLDCSTES-KFVETFRGMGKTSENGFEVWLHEDCAVWSNDIHLIGAHV
B4J9G8 (1119-1356)   QDPLDCSHET-KFVETFRGMSKTSEHGYEIWMHEDCAVWANDIQLIGAHV
B4MYL6 (1108-1349)   ADPLDYSQEN-KLVETFRGMCKTSEHGYEIWLHEDCAIWTNDIQLIGAHV
B3MHU9 (1048-1286)   MDPLDCSHET-KFVETFRGMGKTSEHGYEVWLHEDCAVWSNDIHLIGAHV

                                                 "PHD"
                                  5           6                    7
Q9UGU0 (1690-1939)   YGLQEALEIAREM--KCS-------HCQEAG-------------ATLGCY
G3QDX4 (1690-1939)   YGLQEALEIAREM--KCS-------HCQEAG-------------ATLGCY
H2QLT9 (1692-1941)   YGLQEALEIAREM--KCS-------HCQEAG-------------ATLGCY
H9FZ14 (1690-1939)   YGLQEALEIAREM--KCS-------HCQEAG-------------ATLGCY
G1S175 (1688-1939)   YGLQEALEIAREMPRSC--------HCTPAW-------------ATDRDL
H0XML0 (1702-1951)   YGLQEALEIAREM--KCS-------HCQEAG-------------ATLGCY
I3NC70 (1718-1966)   YGLQEALEIAREM--KCS-------HCQEAG-------------ATLGCY
F7CRS8 (1718-1967)   YGLQEALEIAREM--KCS-------HCQEAG-------------ATLGCY
E2RT08 (1689-1938)   YGLQEALEIAREM--KCS-------HCQEAG-------------ATLGCY
G1P5A9 (1705-1953)   YGLQEALEIAREM--KCS-------HCQEAG-------------ATLGCY
E1B8T3 (1699-1947)   YGLQEALEIAREM--KCS-------HCQEAG-------------ATLGCY
G3U8A2 (1705-1954)   YGLQEALEIAREM--KCS-------HCQEAG-------------ATLGCY
G1LSB5 (1679-1927)   YGLQEALEIAREM--KCS-------HCQEAG-------------ATLGCY
Q9EPQ8 (1718-1962)   YGLQEALEIAREM--KCS-------HCQEAG-------------ATLGCY
G1SYZ5 (1632-1880)   YGLQEALEIAREM--KCS-------HCQEAG-------------ATLGCY
H0VVM3 (1651-1885)   YGLQEALEIAREM--KCS-------HCQEAG-------------ATLGCY
F6Q9R3 (1692-1946)   YGLQEALEIAREM--KCS-------HCQEPG-------------ATLGCY
G3WIE0 (1668-1874)   YGLQEALEIAREM--KCS-------HCQEPG-------------ATLGCY
H0ZHA4 (1664-1913)   YGLQEAVEIAREM--KCS-------HCQEPG-------------ATLGCY
E1BXI6 (1671-1920)   YGLQEAVEIAREM--KCS-------HCQEPG-------------ATLGCY
G1NHL2 (1672-1921)   YGLQEAVEIAREM--KCS-------HCQEPG-------------ATLGCY
G1KC34 (1689-1937)   YGLQEAVEIAKEM--KCS-------HCQEPG-------------ATLGCY
F7BZK2 (1597-1840)   YGLREAVDIAREM--KCS-------HCQETG-------------ATLGCY
I3JNR0 (1311-1549)   YGLQEALDGARET--SCS-------YCEMVG-------------STLGCY
D3ZG21 (1722-1965)   YGLQEALEIAREM--KCS-------HCQEAG-------------ATLGCY
G7PFP9 (1725-1980)   YGLQEALEIAREM--KCS-------HCQEAG-------------ATLGCY
H2P4M2 (1692-1935)   YGLQEALEIAREM--KCS-------HCQEAG-------------ATLGCY
H2ZXL0 (1628-1862)   YGLQEAVEVAREM--RCS-------YCQEIG-------------ATLGCF
G3I0E6 (721-964)     YGLQEALEIAREM--KCS-------HCQEAG-------------ATLGCY
G5BPR9 (1037-1303)   YGLQEALEIAREM--KCS-------HCQEAG-------------ATLGCY
Q4SCG6 (1995-2332)   YGLQEALDGARET--CCS-------YCEMVG-------------STLGCY
E7FE16 (2019-2336)   YGLQEALDGARDA--SCS-------HCEMAG-------------STLGCY
F1SRF7 (1698-1893)   YGLQEALEIAREM-------------------------------------
```

```
H9KVS1(1635-1821)  YGLQEALEIAREM-------------------------------------
Q7Z5J4(1672-1906)  FGLQEAMKVAVDM--MCS-------SCQEA-------------GATIGCC
H2QCD6(1667-1901)  FGLQEAMKVAVDM--MCS-------SCQEA-------------GATIGCC
F7E488(1674-1909)  FGLQEAMKVAVDM--TCS-------SCQEA-------------GATIGCC
H2NSX0(1678-1908)  FGLQEAMKVAVDM--TCS-------SCQEA-------------GATIGCC
G1RTB7(1668-1903)  FGLQEAMKVAVDM--TCS-------SCQEA-------------GATIGCC
E1B9X1(1644-1885)  FGLQEAMKVALDM--TCS-------SCQEA-------------GATIGCC
D4A4Z4(1638-1887)  FGLQEAMKVAVDM--PCT-------SCHESG-------------ATISCS
Q61818(1640-1889)  FGLQEAMKVAVDM--PCT-------SCHEPG-------------ATISCS
G1LF06(1637-1873)  FGLQEAMKVAVDM--ACF-------SCQEA-------------GATIGCC
G3QJR9(1645-1879)  FGLQEAMKVAVDM--MCS-------SCHEA-------------GATIGCC
G3TJ97(1586-1821)  FGLQEAMKVAMDM--TCS-------SCQEAG-------------ATIGCC
H0V1B2(1316-1545)  FGLQEAMKVAVDM--TCS-------SCQEAG-------------ATIGCS
I3N2I1(1670-1914)  FGLQEAMKVAVDM--TCS-------SCQEAG-------------ATIGCS
F7DZK3(1652-1884)  FGLQEAMKVAVDM--TCS-------SCQEA-------------GATIGCC
E1BXD2(1385-1610)  YGLQEAVKAAADLGKKCS-------SCQQAG-------------ATT-AG
G3PEF0(1592-1819)  YGLREAANNSAQT--SCY-------KCQIVG-------------ASLNCC
H3D328(1531-1756)  YGLKEAANKSAQT--SCY-------KCQIVG-------------ASLSCC
E7F726(1703-1936)  FGLTEAVQKAAHA--KCS-------GCQGEG-------------ASVCCG
C3Z7L5(1697-1932)  HGLEDAIKEARQH--SCS-------ECETVG-------------ATLGCG
E2BIR7(1229-1457)  TGLQEAVWDAAKS--ICC-------SCGLTG-------------ANIGCI
E2B1Q5(1212-1434)  TGLQEAVWDAAKS--ICD-------SCGLTG-------------ANIGCV
F4W5Y0(1113-1335)  TGLQEAVWDAAKS--VCD-------SCGLTG-------------ANIGCV
H9HN29(1142-1364)  TGLQEAVWDAAKS--VCD-------SCGLTG-------------ANIGCV
H9K8F1(1148-1369)  TGLQEAVWDAAKS--ICD-------SCGLTG-------------ANIGCV
G1MZ73(1318-1538)  YGLQEAVKAAADL--KCS-------SCQQAG-------------ATVGCC
F6TA33(1577-1806)  YGINEAIQMAAAG--LCP-------KCQRPG-------------ATVGCS
G3X2Q6(1102-1337)  FGLQEAMKTAADV--RCS-------SCQQVG-------------ATLGCC
G5BMW5(1673-1926)  FGLQEAMKVAVDM--VRG------QPSGVGT-----------QLLGLGPS
F6R4X5(1594-1827)  FGLQEAMKTAADV--VRPSLG---APALAP--------------SSLDAV
F7B730(1669-1926)  FGLQEAMKVAMDM--HGGAV---VLAPGGISLLSLRL-----TSLFQGPS
E2RE72(1543-1784)  FGLQEAMKVAVDM--ESG---MPLMGCGRASVQATDF-----VSLFRGPP
H0Y065(1671-1929)  FGLQEAMKVAIDM--VRGQP------CGVAGDSSK-PRLRQKDLLETR-S
H9G548(1666-1853)  YGLQEAIKMAANL--VRP--------------------------------
H3AX41(1594-1783)  YGLKEAIETAITV-------------------------------VSLN--
G1NTH3(1623-1838)  FGLQEAMKVALDM--TCS-------SCQEAG-------------ATIGCC
G3HHP8(1539-1764)  FGLQEAMKVAVDM--PCS-------SCHEAG-------------ATISCS
Q6AWG9(967-1203)   NGLDAAVWDSTRY--QCV-------LCQQTG-------------ASICCF
Q16VV4(907-1120)   VGLEAAIWNCCRH--QCR-------ICSHHG-------------ATVGCL
B3NLN9(1085-1322)  NGLDAAVWESTRY--QCV-------LCQQTG-------------ASICCF
B0W5K6(960-1174)   VGLEAAIWNCCRH--PCR-------ICGHSG-------------AVVSCL
A7UTC0(931-1162)   VGLEAAIWNCCRH--QCQ-------LCRNYG-------------AVLSCL
E3XES6(921-1156)   IGLEAAIWNCCRH--RCQ-------LCSQYG-------------AVVCCL
B4HNB7(1026-1264)  NGLDAAVWDSTRY--QCV-------LCQQTG-------------ASICCF
B4QBU9(866-1104)   NGLDAAVWDSTRY--QCV-------LCQQTG-------------ASICCF
B4J9G8(1119-1356)  NGLDAAVWDSTRY--QCV-------HCAQSG-------------ANVCCF
B4MYL6(1108-1349)  NGLDAAVWDSTRY--QCV-------HCSQTG-------------ANVCCF
B3MHU9(1048-1286)  NGLDAAVWDSTRY--QCV-------LCQQSG-------------ANICCF
```

"PHD"

```
                                      8     9  10
Q9UGU0(1690-1939)  NKG----------------CSFR-YHYPCAIDA-----------------
G3QDX4(1690-1939)  NKG----------------CSFR-YHYPCAIDA-----------------
H2QLT9(1692-1941)  NKG----------------CSFR-YHYPCAIDA-----------------
H9FZ14(1690-1939)  NKG----------------CSFR-YHYPCAVDA-----------------
G1S175(1688-1939)  NGI----------------CHFLGLH--CPVEMDA---------------
H0XML0(1702-1951)  NKG----------------CSFR-YHYPCAIDA-----------------
I3NC70(1718-1966)  NKG----------------CSFR-YHYPCAIDA-----------------
F7CRS8(1718-1967)  NKG----------------CSFR-YHYPCAIDA-----------------
E2RT08(1689-1938)  NKG----------------CSFR-YHYPCAIDA-----------------
G1P5A9(1705-1953)  NKG----------------CSFR-YHYPCAIDA-----------------
E1B8T3(1699-1947)  NKG----------------CSFR-YHYPCAIDA-----------------
G3U8A2(1705-1954)  NKG----------------CSFR-YHYPCAIDA-----------------
G1LSB5(1679-1927)  NKG----------------CSFR-YHYPCAIDA-----------------
Q9EPQ8(1718-1962)  NKG----------------CSFR-YHYPCAIDA-----------------
G1SYZ5(1632-1880)  NKG----------------CSFR-YHYPCAVDA-----------------
H0VVM3(1651-1885)  NKG----------------CSFR-YHYPCAVDA-----------------
F6Q9R3(1692-1946)  NKG----------------CSFR-YHYPCAIDA-----------------
G3WIE0(1668-1874)  NKG----------------CSFR-YHYPCAIDA-----------------
H0ZHA4(1664-1913)  NKG----------------CSFR-YHYPCAIDA-----------------
E1BXI6(1671-1920)  NKG----------------CSFR-YHYPCAIDA-----------------
G1NHL2(1672-1921)  NKG----------------CSFR-YHYPCAIDA-----------------
G1KC34(1689-1937)  NKG----------------CSFR-YHYPCAIDA-----------------
F7BZK2(1597-1840)  NKG----------------CACC-YHFPCAMDS-----------------
I3JNR0(1311-1549)  SKG----------------CTLR-YHYLCAIEA-----------------
D3ZG21(1722-1965)  NKG----------------CSFR-YHYPCAIDA-----------------
G7PFP9(1725-1980)  NKG----------------CSFR-YHYPCAVDAA--------------SV
```

```
H2P4M2(1692-1935)  NKG----------------CSFR-YHYPCAIDA-----------------
H2ZXL0(1628-1862)  SKG----------------CSFR-YHYPCAKEA-----------------
G3I0E6(721-964)    NKG----------------CSFR-YHYPCAIDA-----------------
G5BPR9(1037-1303)  NKG----------------CSFR-YHYPCAIDAGGQLHQAALLLFGLDRR
Q4SCG6(1995-2332)  SKG----------------CTLR-YHYLCAIEA-----------------
E7FE16(2019-2336)  SKG----------------CTLR-YHYICAIDA-----------------
F1SRF7(1698-1893)  --------------------------------------------------
H9KVS1(1635-1821)  --------------------------------------------------
Q7Z5J4(1672-1906)  HKG----------------CLHT-YHYPCASDAG----------------
H2QCD6(1667-1901)  HKG----------------CLHT-YHYPCASDAG----------------
F7E488(1674-1909)  HKG----------------CIHT-YHYPCASDAG----------------
H2NSX0(1678-1908)  HKG----------------CIHT-YHYPCASDAG----------------
G1RTB7(1668-1903)  HKG----------------CIHT-YHYPCASDAG----------------
E1B9X1(1644-1885)  HKG----------------CLHT-YHYPCASDAG----------------
D4A4Z4(1638-1887)  YEG----------------CTHT-YHYPCANDTG----------------
Q61818(1640-1889)  YKG----------------CIHT-YHYPCANDTG----------------
G1LF06(1637-1873)  QKG----------------CTHT-YHYPCASDAG----------------
G3QJR9(1645-1879)  HKG----------------CLHT-YHYPCASDAG----------------
G3TJ97(1586-1821)  HKG----------------CVHT-YHYPCASDAG----------------
H0V1B2(1316-1545)  YKG----------------CIHT-YHYPCASDAG----------------
I3N2I1(1670-1914)  YKG----------------CVHS-YHYPCASDAG----------------
F7DZK3(1652-1884)  QKG----------------CIHT-YHYPCASDAG----------------
E1BXD2(1385-1610)  GRG----------------CPHM-LS---PFPTG----------------
G3PEF0(1592-1819)  WRG----------------CSHK-YHYVCAKEIG----------------
H3D328(1531-1756)  WRG----------------CSHK-YHYVCAKEIG----------------
E7F726(1703-1936)  WKS----------------CTQS-YHYICAKESG----------------
C3Z7L5(1697-1932)  FKG----------------CQLK-YHYVCAVDAG----------------
E2BIR7(1229-1457)  KRG----------------CKAV-SHYPCALTKG----------------
E2B1Q5(1212-1434)  KRG----------------CKAV-THYPCALTKG----------------
F4W5Y0(1113-1335)  KRG----------------CKAV-SHYPCALTKG----------------
H9HN29(1142-1364)  KRG----------------CKAV-SHYPCALTKG----------------
H9K8F1(1148-1369)  KRG----------------CKAV-IHYPCALTKG----------------
G1MZ73(1318-1538)  QKG----------------CPHT-YHYACAVDTG----------------
F6TA33(1577-1806)  HKG----------------CEQS-YHITCAVEAAG---------------
G3X2Q6(1102-1337)  HKG----------------CPQT-YHYACASDTG----------------
G5BMW5(1673-1926)  WKSKFAGKSPP--LYF---C----RRVPAARKPG----------------
F6R4X5(1594-1827)  SPGGRWEGAFLVLTGFQ--AAWD-SVLAQASAPG----------------
F7B730(1669-1926)  WPG----------------CPLL-AMSTPSSWARYV-PAAKKPG------
E2RE72(1543-1784)  WP-----------------CPAV-PAASLSDSWAGPVPAAKKPG------
H0Y065(1671-1929)  WRL----------------CPSS-AVHLPPSLFIRHVPVVRNPG------
H9G548(1666-1853)  --------------------------------------------------
H3AX41(1594-1783)  -------------------C------------------------------
G1NTH3(1623-1838)  HKG----------------CAHT-YHYPCASDAG----------------
G3HHP8(1539-1764)  YKG----------------CIHT-YHYPCANDTG----------------
Q6AWG9(967-1203)   QRC----------------CKAA-AHVPCGRSAN----------------
Q16VV4(907-1120)   QRG----------------CSEE-AHVVCARRND----------------
B3NLN9(1085-1322)  QRC----------------CKAA-AHVPCARSAN----------------
B0W5K6(960-1174)   RRG----------------CNAE-AHVVCARKHD----------------
A7UTC0(931-1162)   HQG----------------CHAK-AHFICAHKQH----------------
E3XES6(921-1156)   RQG----------------CTAK-AHFICAHKQN----------------
B4HNB7(1026-1264)  QRC----------------CKAA-AHVPCARSDN----------------
B4QBU9(866-1104)   QRC----------------CKAA-AHVPCARSDN----------------
B4J9G8(1119-1356)  QRA----------------CKAA-AHVPCARVAS----------------
B4MYL6(1108-1349)  QRS----------------CKAT-AHVPCARSAN----------------
B3MHU9(1048-1286)  QRC----------------CKAP-AHVPCARSAN----------------
```

"PHD"

11 12

```
Q9UGU0(1690-1939)  ---------DCLLHEENFSVRCPKHKPPLPCP
G3QDX4(1690-1939)  ---------DCLLHEENFSVRCPKHKPPLPCP
H2QLT9(1692-1941)  ---------DCLLHEENFSVRCPKHKPPLPCP
H9FZ14(1690-1939)  ---------DCLLHEENFSVRCPKHKPPLPCP
G1S175(1688-1939)  ---------YCLLHEENFSVRCPKHKPPLPCP
H0XML0(1702-1951)  ---------DCLLHEENFSVRCPKHKPPLPCP
I3NC70(1718-1966)  ---------DCLLHEENFSVRCPKHKPPLPCP
F7CRS8(1718-1967)  ---------DCLLHEENFSVRCPKHKPPLPCP
E2RT08(1689-1938)  ---------DCLLHEENFSVRCPKHKPPLPFP
G1P5A9(1705-1953)  ---------DCLLHEENFSVRCPKHKPPLPCP
E1B8T3(1699-1947)  ---------DCLLHEENFSVRCPKHKPPLPCP
G3U8A2(1705-1954)  ---------DCLLHEENFSLRCPKHKPPLPCP
G1LSB5(1679-1927)  ---------DCLLHEENFSVRCPKHKPPLPFP
Q9EPQ8(1718-1962)  ---------DCLLHEENFSVRCPKHK----CP
G1SYZ5(1632-1880)  ---------DCLLHEENFSVRCPKHKPPLPCP
H0VVM3(1651-1885)  ---------DCLLHEENFSVRCPKHKPPLPCP
F6Q9R3(1692-1946)  ---------DCLLNEENFSVRCPKHKPPLPCS
G3WIE0(1668-1874)  ---------DCLLNEENFSVRCPKHKPPLPCS
H0ZHA4(1664-1913)  ---------DCLLNEENFSVRCPKHKPLLPCS
```

```
E1BXI6(1671-1920)  ---------DCLLNEENFSVRCPKHKPLLPCS
G1NHL2(1672-1921)  ---------DCLLNEENFSVRCPKHKPLLPCS
G1KC34(1689-1937)  ---------DCLLNEENFSVRCPKHKPLLPCS
F7BZK2(1597-1840)  ---------ECLLNEENFSVRCPKHKIPISSS
I3JNR0(1311-1549)  ---------DCSLNEDNFSLRCPKHKFVFFPP
D3ZG21(1722-1965)  ---------DCLLHEENFSVRCPKHK------
G7PFP9(1725-1980)  GETVKKNKRDCLLHEENFSVRCPKHK------
H2P4M2(1692-1935)  ---------DCLLHEENFSVRCPKHK------
H2ZXL0(1628-1862)  ---------ECFLSEENFSMRCRKHK------
G3I0E6(721-964)    ---------DCLLHEENFSVRCPKHK------
G5BPR9(1037-1303)  LPVQRCQSPDCLLHEENFSVRCPKHK------
Q4SCG6(1995-2332)  ---------DCSLNEDNFSLRCPKHK------
E7FE16(2019-2336)  ---------DCSLNEDNFSLRCPKHK------
F1SRF7(1698-1893)  --------------------------------
H9KVS1(1635-1821)  --------------------------------
Q7Z5J4(1672-1906)  ----------CIFIEENFSLKCPKHKR-LP--
H2QCD6(1667-1901)  ----------CIFIEENFSLKCPKHKR-LP--
F7E488(1674-1909)  ----------CIFIEENFSLKCPKHKR-LPL-
H2NSX0(1678-1908)  ----------CIFIEENFSLKCPKHKR-LPL-
G1RTB7(1668-1903)  ----------CIFIEENFSLKCPKHKR-LPL-
E1B9X1(1644-1885)  ----------CIFIEENFSLKCPKHKR-LPL-
D4A4Z4(1638-1887)  ----------CTFIEENFTLKCPKHKR-LPL-
Q61818(1640-1889)  ----------CTFIEENFTLKCPKHKR-LPL-
G1LF06(1637-1873)  ----------CILIEENFSLKCPKHKRSLPA-
G3QJR9(1645-1879)  ----------CIFIEENFSLKCPKHKR-LP--
G3TJ97(1586-1821)  ----------CIFMEENFSLKCPKHKR-LPL-
H0V1B2(1316-1545)  ----------CVFIEENFSLKCPKHKR-LPL-
I3N2I1(1670-1914)  ----------CVFIEENFSLKCPKHK------
F7DZK3(1652-1884)  ----------CIFIEENFSLKCPKHK------
E1BXD2(1385-1610)  ----------CLLTEESFSLRCPKHKVS-PE-
G3PEF0(1592-1819)  ----------CTFHEDDFSIKCPKHEQL-P--
H3D328(1531-1756)  ----------CTFHEDDFSIKCPKHEVIK---
E7F726(1703-1936)  ----------CTFEEETFSIRCPKHKVSV---
C3Z7L5(1697-1932)  ----------CCLSEENFSLMCYQHKHKTIRF
E2BIR7(1229-1457)  ----------WHLDTNQYIPKCNLHRVT----
E2B1Q5(1212-1434)  ----------WHLDTNQYIPKCNLHRVT----
F4W5Y0(1113-1335)  ----------WHLDTNQYIPKCNLHRVT----
H9HN29(1142-1364)  ----------WHLDTNQYIPKCNLHRVT----
H9K8F1(1148-1369)  ----------WHLDINQYIPKCNLHRIT----
G1MZ73(1318-1538)  ----------CLLTEECFSLRCPKHKDI-PL-
F6TA33(1577-1806)  ----------CLLSEENFSLRCPKHKVRKPV-
G3X2Q6(1102-1337)  ----------CILVEENFSLKCPKHKLI-P--
G5BMW5(1673-1926)  --------PPLGAPTKDASTPTTTHV---PVM
F6R4X5(1594-1827)  --------PPLAAATKDVLKPTTTHV---PVT
F7B730(1669-1926)  --------PPLGAATKDASTPITTRV---PAM
E2RE72(1543-1784)  --------PPSGAATKDAPTPTITRV---PAM
H0Y065(1671-1929)  --------PPSGAATKDVSTLTITRV---PVM
H9G548(1666-1853)  --------------------------------
H3AX41(1594-1783)  --------------------------------
G1NTH3(1623-1838)  --------------------------------
G3HHP8(1539-1764)  --------------------KS----------
Q6AWG9(967-1203)   ----------WSLSEEDRKVYCHLHRHE-PGV
Q16VV4(907-1120)   ----------WELS-DEFKSHCEKHSKTDSS-
B3NLN9(1085-1322)  ----------WSLSEEDRKVYCQLHRHQ-PEI
B0W5K6(960-1174)   ----------WELS-DEFKAHCKDHSEGGDS-
A7UTC0(931-1162)   ----------WKLT-EDFQSFCDRHSGHSGGS
E3XES6(921-1156)   ----------WKLT-DEYQAFCEVHAMKEPGS
B4HNB7(1026-1264)  ----------WSLSEEDRKVYCQLHRHEPDVV
B4QBU9(866-1104)   ----------WSLSEEDRKVYCQLHRHEPDVV
B4J9G8(1119-1356)  ----------WSLSEQDRKVYCQLHAPQKAKE
B4MYL6(1108-1349)  ----------WHLNEHERKVFCQLHGPSEGSH
B3MHU9(1048-1286)  ----------WSLSEEDRKVHCQLHSGESGVA
```

Figure S1
