# Supplementary material for: A Phylogenetic Study of SPBP and RAI1: Evolutionary Conservation of Chromatin Binding Modules
Source: PLoS One. 2013 Oct 18;8(10):e78907. doi: 10.1371/journal.pone.0078907 (PMC3799622; doi:10.1371/journal.pone.0078907)
Supplement: Table S1 — cDNA constructs made in this study. (DOCX) [file pone.0078907.s002.docx]

**Table S1.**

**____________________________________________________________________________________________________________________**

**Name Description**

pGBKT7-SPBP(ePHD3-12) ePHD domain of SPBP (aa 1837-1960), long splice variant, lacking the two most N-terminal

zinc-ligands. PCR amplified using primer 1 and 2, and ligated into end-filled *Nco*I site of pGBKT7.

pGBKT7-SPBP(ePHD5-12) ePHD domain of SPBP lacking the four N-terminal putative zinc ligands (aa 1870-1960).

PCR amplified using primer 1 and 4, and ligated into *EcoR*I and *BamH*I sites of pGBKT7.

pENTR-SPBP(F box) Region of human SPBP containing F box (aa 1678-1741), PCR amplified using primer 3 and 5,

and ligated into *Dra*I and *EcoR*I sites of pENTR1A.

pENTR-RAI1(F box) Region of human RAI1 containing F box, PCR amplified using primer 6 and 7, and ligated into

the *Dra*I and *EcoR*I sites of pENTR1A.

pENTR-MLL1(ePHD/ADD) ePHD/ADD domain of human MLL1, PCR amplified using primer 8 and 9 on human placenta Marathon

Ready cDNA (Clontech), and ligated into the *Dra*I and *EcoR*V sites of pENTR1A.

pENTR-MLL2(ePHD/ADD) ePHD/ADD domain of human MLL2, PCR amplified using primer 10 and 11, on human placenta Marathon

Ready cDNA (Clontech), and ligated into the *Dra*I and *EcoR*I sites of pENTR1A.

pENTR-MLL3(ePHD/ADD) ePHD/ADD domain of human MLL3, PCR amplified using primer 12 and 13 on human placenta

Marathon Ready cDNA (Clontech), and ligated into the *Dra*I and *EcoR*I sites of pENTR1A.

pDONR221-RAI1 (Δ1523-1627) RAI1 without novel nucleosome binding domain. Made by a PCR deletion strategy using primers 21 and 22.

The PCR product was digested by *ECORI* and ligated.

pENTR-SPBP(ePHD)-CCLA Primer 14. Template: pENTR-SPBP(ePHD)

pENTR-SPBP(ePHD)-ACLA Primer 15 Template: pENTR-SPBP(ePHD)

pENTR-SPBP(ePHD)-CALC Primer 16 Template: pENTR-SPBP(ePHD)

pDONR207-RAI1(ePHD3-12) Truncated ePHD domain of human RAI1, PCR amplified using primer 17 and 18.

pDONR221-RAI1(1523-1627) Novel nucleosome binding region of human RAI1. PCR amplified using primers 19 and 20.

**____________________________________________________________________________________________________________________**
