# Supplementary material for: A Phylogenetic Study of SPBP and RAI1: Evolutionary Conservation of Chromatin Binding Modules
Source: PLoS One. 2013 Oct 18;8(10):e78907. doi: 10.1371/journal.pone.0078907 (PMC3799622; doi:10.1371/journal.pone.0078907)
Supplement: Table S2 — Primers used in this study. (DOCX) [file pone.0078907.s003.docx]

**Table S2.**

**No. Name Sequence**

1 hSPBP5176-3’ 5’-CTGGATCCTGTGCTTGCTGTCCTTTCCATT-3’

2 hSPBP5176-3’ 5’-CTGGATCCTGTGCTTGCTGTCCTTTCCATT-3’

3 hSPBP5166-5’ 5’-GAAAGCAAGGCGCTCCCGGCC-3’

4 hSPBP5742-5’ 5’-CGAATTCGGCAGGCTCTATGGCCTGCAGGAAG-3’

5 hSPBP5358-3’ 5’-CGAATTCCCTCTTAGGAGGTGGATTCTTGC-3’

6 RAI1Stopp-3’ 5’-CGAATTCGTTGGGGTGGATTACTACGGCAG-3’

7 RAI1F box-3’ 5’-CGAATTCTTGAGTTTTGGCTTCTTTTTGGGGAG-3’

8 MLL1ePHD-5’ 5’-GATAGGAGTCGAGAAGACAGTCCAG-3’

9 MLL1ePHD-3’ 5’-CGAATTCCATTCTCAGGAACCACTTCGCC-3’

10 MLL2ePHD-5’ 5’-GGCAAGGATCCGGCTGCCTTCTC-3’

11 MLL2ePHD-3’ 5’-CGAATTCCATCGGGGTTCACAATTTCCTTGC-3’

12 MLL3PHD-5’ 5’-GGCACAGCCTTGCGACCTGACAAG-3’

13 MLL3PHD-3’ 5’-CGAATTCGCTCAATGTAGACCCGCCGGAAG-3’

14 SPBP-F box CCLA-5’ 5’-GGTTTGCTGTCTGGCTGGCAAGTGGGCCAG-3’

15 SPBP-F box ACLA-5’ 5’-CCCACTTGCCAGCCAGACAGGCAACCAGGTGCCC-3’

16 SPBP-F box CALC-5’ 5’-GCACCTGGTTTGCGCTCTGTGTGGCAAGTGG-3’

17 GateRai1ZNF-5’ 5’GGGGACAAGTTTGTACAAAAAAGCAGGCTTGTGCAGCAAGGAGGCTCCG-3’

18 GateRai1ZNF-3’ 5’-GGGGACCACTTTGTACAAGAAAGCTGGGTGTGGATTACTACGGCAGC-3’

19 RAI1 (1523-1627)-5’ 5’-GGGGCAACTTTGTACAAAAAAGCAGGCTCCACCCAGAAACAGCCAGGCAC-3’

20 RAI1 (1523-1627)-3’ 5’-GGGGACCACTTTGTACAAGAAAAGCTGGGTCTACTTAAGGCTTCCTGTGGGGCTT-3’

21 RAI1 (Δ1523-1627)-5’ 5’-CCGGAATTCTCCTCCTCTGCCTCCTCTT-3’

22 RAI1 (Δ1523-1627)-3’ 5’-CCGGAATTCTGCCCTTGTCTGGGGCTG-3’

|  |
| --- |
