# Supplementary material for: A Phylogenetic Study of SPBP and RAI1: Evolutionary Conservation of Chromatin Binding Modules
Source: PLoS One. 2013 Oct 18;8(10):e78907. doi: 10.1371/journal.pone.0078907 (PMC3799622; doi:10.1371/journal.pone.0078907)
Supplement: Table S3 — The ePHD/ADD domain of SPBP and RAI1 in different species. (DOCX) [file pone.0078907.s004.docx]

**Table S3.**

| **Uniprot accession number** | **Species name** | **Protein name** | **Amino acids positions** |
| --- | --- | --- | --- |
| **Mammalia** | | | |
| Q9UGU0 | H.sapiens | TCF20 | (1690-1939) |
| G3QDX4 | G.gorilla | TCF20 | (1690-1939) |
| H2QLT9 | P.troglodytes | ENSG00000100207 | (1692-1941) |
| H9FZ14 | M.mulatta | TCF20 | (1690-1939) |
| G1S175 | N.leucogenys | TCF20 | (1688-1939) |
| H0XML0 | O.garnettii | TCF20 | (1702-1951) |
| I3NC70 | S.tridecemlineatus | TCF20 | (1718-1966) |
| F7CRS8 | E.caballus | TCF20 | (1718-1967) |
| E2RT08 | C.familiaris | TCF20 | (1689-1938) |
| G1P5A9 | M.lucifugus | TCF20 | (1705-1953) |
| E1B8T3 | B.taurus | TCF20 | (1699-1947) |
| G3U8A2 | L.africana | TCF20 | (1705-1954) |
| G1LSB5 | A.melanoleuca | TCF20 | (1679-1927) |
| Q9EPQ8 | M.musculus | TCF20 | (1718-1962) |
| G1SYZ5 | O.cuniculus | TCF20 | (1632-1880) |
| H0VVM3 | C.porcellus | TCF20 | (1651-1885) |
| F6Q9R3 | M.domestica | TCF20 | (1692-1946) |
| G3WIE0 | S.harrisii | TCF20 | (1668-1874) |
| G3I0E6 | C.griseus | I79_016828 | (721-964) |
| G5BPR9 | H.glaber | GW7_19457 | (1037-1303) |
| D3ZG21 | R.norvegicus | TCF20 | (1722-1965) |
| G7PFP9 | M.fascicularis | EGM_02755 | (1725-1980) |
| H2P4M2 | P.abelii | TCF20 | (1692-1935) |
| F1SRF7 | S.scrofa | TCF20 | (1698-1893) |
| H9KVS1 | C.jacchus | LOC100415239 | (1635-1821) |
| Q7Z5J4 | H.sapiens | RAI1 | (1672-1906) |
| H2QCD6 | P.troglodytes | ENSG00000108557 | (1667-1901) |
| F7E488 | M.mulatta | RAI1 | (1674-1909) |
| H2NSX0 | P.abelii | LOC100438433 | (1678-1908) |
| G1RTB7 | N.leucogenys | RAI1 | (1668-1903) |
| E1B9X1 | B.taurus | RAI1 | (1644-1885) |
| D4A4Z4 | R.norvegicus | RAI1 | (1638-1887) |
| Q61818 | M.musculus | RAI1 | (1640-1889) |
| G1LF06 | A.melanoleuca | RAI1 | (1637-1873) |
| G3QJR9 | G.gorilla | RAI1 | (1645-1879) |
| G3TJ97 | L.africana | RAI1 | (1586-1821) |
| H0V1B2 | C.porcellus | RAI1 | (1316-1545) |
| I3N2I1 | S.tridecemlineatus | RAI1 | (1670-1914) |
| F7DZK3 | E.caballus | RAI1 | (1652-1884) |
| G3X2Q6 | S.harrisii | RAI1 | (1102-1337) |
| G5BMW5 | H.glaber | GW7_07648 | (1673-1926) |
| F6R4X5 | M.domestica | RAI1 | (1594-1827) |
| F7B730 | C.jacchus | RAI1 | (1669-1926) |
| E2RE72 | C.familiaris | RAI1 | (1543-1784) |
| H0Y065 | O.garnettii | RAI1 | (1671-1929) |
| G1NTH3 | M.lucifugus | RAI1 | (1623-1838) |
| G3HHP8 | C.griseus | I79_010154 | (1539-1764) |
| **Aves (Birds)** | | | |
| H0ZHA4 | T.guttata | TCF20 | (1664-1913) |
| E1BXI6 | G.gallus | TCF20 | (1671-1920) |
| G1NHL2 | M.gallopavo | TCF20 | (1672-1921) |
| G1MZ73 | M.gallopavo | RAI1 | (1318-1538) |
| E1BXD2 | G.gallus | RAI1 | (1385-1610) |
| **Reptilla** | | | |
| G1KC34 | A.carolinensis | TCF20 | (1689-1937) |
| H9G548 | A.carolinensis | RAI1 | (1666-1853) |
| **Amphibia** | | | |
| F7BZK2 | X.tropicalis | TCF20 | (1597-1840) |
| F6TA33 | X.tropicalis | RAI1 | (1577-1806) |
| **Sarcopterygii** | | | |
| H2ZXL0 | L.chalumnae | TCF20 | (1628-1862) |
| H3AX41 | L.chalumnae | RAI1 | (1594-1783) |
| **Actinopterygii** | | | |
| I3JNR0 | O.niloticus | TCF20 | (1311-1549) |
| Q4SCG6 | T.nigroviridis | GSTENG00020500001 | (1995-2332) |
| E7FE16 | D.rerio | si:zfos-1697h8.3 | (2019-2336) |
| G3PEF0 | G.aculeatus | RAI1 | (1592-1819) |
| H3D328 | T.nigroviridis | RAI1 | (1531-1756) |
| E7F726 | D.rerio | RAI1 | (1703-1936) |
| **Leptocardii** | | | |
| C3Z7L5 | B.floridae | BRAFLDRAFT_69184 | (1697-1932) |
| **Insecta** | | | |
| E2BIR7 | H.saltator | EAI_04173 | (1229-1457) |
| E2B1Q5 | C.floridanus | EAG_02123 | (1212-1434) |
| F4W5Y0 | A.echinatior | G5I_00830 | (1113-1335) |
| H9HN29 | A.cephalotes |  | (1142-1364) |
| Q6AWG9 | D.melanogaster | CG5098 | (967-1203) |
| Q16VV4 | A.aegypti | AAEL009427 | (907-1120) |
| B3NLN9 | D.erecta | GG21841 | (1085-1322) |
| B0W5K6 | C.quinquefasciatus | CPIJ002321 | (960-1174) |
| A7UTC0 | A.gambiae | AGAP005259 | (931-1162) |
| E3XES6 | A.darlingi | AND_22215 | (921-1156) |
| B4HNB7 | D.sechellia | GM21842 | (1026-1264) |
| B4QBU9 | D.simulans | GD11336 | (866-1104) |
| B4J9G8 | D.grimshawi | GH21445 | (1119-1356) |
| H9K8F1 | A.mellifera | LOC727236 | (1148-1369) |
| B4MYL6 | D.willistoni | GK22244 | (1108-1349) |
| B3MHU9 | D.ananassae | GF11689 | (1048-1286) |
