# Supplementary material for: A Phylogenetic Study of SPBP and RAI1: Evolutionary Conservation of Chromatin Binding Modules
Source: PLoS One. 2013 Oct 18;8(10):e78907. doi: 10.1371/journal.pone.0078907 (PMC3799622; doi:10.1371/journal.pone.0078907)
Supplement: Table S4 — Novel nucleosome-binding region of SPBP and RAI1 in different species. (DOCX) [file pone.0078907.s005.docx]

**Table S4.**

| **Uniprot accession number** | **Species name** | **Protein name** | **Amino acids positions** |
| --- | --- | --- | --- |
| Q9UGU0 | H.sapiens | TCF20 | **(1536-1666)** |
| E1BXI6 | G.gallus | TCF20 | **(1512-1647)** |
| G1KC34 | A.carolinensis | TCF20 | **(1526-1665)** |
| F7BZK2 | X.tropicalis | TCF20 | **(1424-1574)** |
| H2ZXL0 | L.chalumnae | TCF20 | **(1487-1605)** |
| E7FE16 | D.rerio | si:zfos-1697h8.3 | **(1781-1970)** |
| Q7Z5J4 | H.sapiens | RAI1 | **(1508-1632)** |
| E1BXD2 | G.gallus | RAI1 | **(1247-1369)** |
| H9G548 | A.carolinensis | RAI1 | **(1525-1648)** |
| F6TA33 | X.tropicalis | RAI1 | **(1435-1555)** |
| H3AX41 | L.chalumnae | RAI1 | **(1454-1575)** |
| E7F726 | D.rerio | Uncharacterized protein | **(1577-1685)** |
